# Embedding Pd into SnO_2_ drastically enhances gas sensing†

**DOI:** 10.1039/d3na00558e

**Published:** 2024-01-31

**Authors:** Katarzyna Jabłczyńska, Alexander Gogos, Christian M. P. Kubsch, Sotiris E. Pratsinis

**Affiliations:** a Particle Technology Laboratory, Institute of Energy and Process Engineering, Department of Mechanical and Process Engineering, ETH Zurich CH-8092 Zurich Switzerland pratsinis@ethz.ch; b Faculty of Chemical and Process Engineering, Warsaw University of Technology 00-645 Warsaw Poland; c Particles-Biology Interactions, Department of Materials Meet Life, Swiss Federal Laboratories for Materials Science and Technology (Empa) CH-9014 St. Gallen Switzerland; d Nanoparticle Systems Engineering Laboratory, Institute of Energy and Process Engineering, Department of Mechanical and Process Engineering, ETH Zurich CH-8092 Zurich Switzerland

## Abstract

Combustion aerosol processes can uniquely embed noble metals into semiconducting particles. Here, monocrystalline SnO_2_ particles embedded with Pd and/or PdO_*x*_ were made by flame spray pyrolysis (FSP) of appropriate precursors through microexplosions by droplet-to-particle conversion as the crystal size was proportional to the cube root of precursor solution concentration, *C*. These particles were air-annealed and leached with nitric acid for removal of metallic Pd from their surface. The SnO_2_ crystal size varied from 11 to 24 nm and was in close agreement with the primary particle size determined by nitrogen adsorption. The embedded fraction of Pd ranged from about 30 to 80% of the nominal Pd-content. This was achieved by judiciously varying the *C*, Pd content and the ratio of precursor solution to dispersion oxygen flowrates during FSP. The response of sensors made by doctor blading films of such particles to 1 ppm of acetone and CO was evaluated at 350 °C and 50% relative humidity. Embedding Pd/PdO_*x*_ into SnO_2_ significantly increased the sensor response: 2–6 times over that of pure or conventionally-made Pd-containing SnO_2_ sensors at low nominal Pd-contents (0.2 mol%). For higher ones (*i.e.* 1 mol% Pd), the sensor response was enhanced by up to two orders of magnitude. This is attributed to Pd atoms in the SnO_2_ lattice near the particle surface and/or Pd/PdO_*x*_ clusters acting as nanoelectrodes into SnO_2_ films and altering their transducing properties as shown by high resolution electron microscopy, XPS and baseline resistance measurements of pure and Pd-embedded SnO_2_ sensing films.

## Abstract 2

The sensitivity of SnO_2_ increases by up to 2-orders of magnitude when embedding Pd atoms or clusters into its matrix.

## Graphical abstract

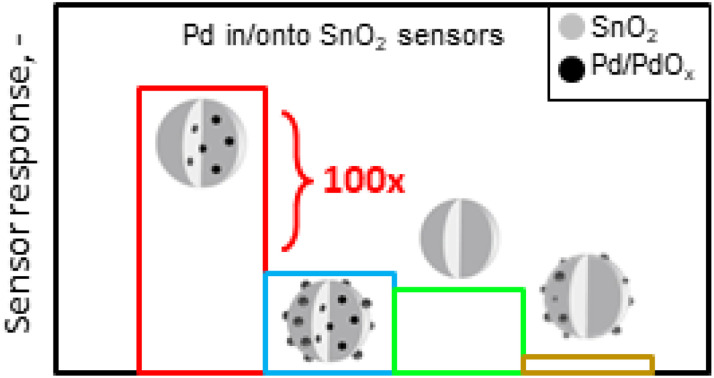

## Introduction

Gas sensors are used widely for detection of drunk driving and monitoring compliance of automotive emissions as well as to prevent gas explosions and poisoning in mines. Due to their low cost and portability, they are most promising for monitoring air quality outdoors (*e.g.* NO_2_)^1^ or indoors (*e.g.* formaldehyde),^2^ food quality (*e.g.* methanol in alcoholic beverages),^3^ for medical diagnostics (*e.g.* diabetes or inflammatory markers such as NO for asthma)^4^ or monitoring fat-burning from breath acetone.^5^ What links these applications is the requirement to selectively measure very low, sometimes at few ppb, concentrations of specific analytes. Therefore, there is a clear need for highly sensitive materials driving the search of new chemical compositions and novel material structures (or morphologies).

Standard chemiresistive gas sensors consist of a functional film of semiconducting nanoparticles, deposited between a pair of electrodes. When that film is exposed to analyte gas it serves as the catalyst for the reaction between adsorbed oxygen species and analyte on the film surface.^6^ This reaction alters the concentration of charge carriers in the film and can be measured as a change of its resistance. Therefore, a good sensor, among other features, should give the highest possible response, *i.e.* a change in resistance in relation to the baseline in clean air.

Tin oxide is the most common gas sensor. Doping it with noble metals (NM) is frequently used to enhance its sensitivity.^7^ In contrast to most methods for preparation of SnO_2_ with NM (impregnation,^8^ reverse micelles,^9^ ball mill mixing^10^ and photodeposition^11^) in which SnO_2_ particles are only externally decorated with NM clusters, flame spray pyrolysis (FSP) uniquely allows placing NM on the surface and inside the sensing nanoparticles.^12^ Recently it was shown that solely embedding palladium (Pd) or PdO into SnO_2_ increased significantly the sensor response to CO, acetone and ethanol over that from pure and Pd-decorated SnO_2_ made, for example, by Pd photodeposition.^13^

Here, this discovery is explored systematically by varying the embedded Pd-content and SnO_2_ crystal size by closely controlling the FSP synthesis of such particles. Palladium is removed from the surface of SnO_2_ by leaching and the effect of embedded Pd fraction on sensor response to 1 ppm of inorganic (CO) and organic vapors (acetone) at 350 °C and 50% relative humidity is investigated focusing on the sensitivity of such materials as their selectivity can be enhanced readily by filters.^14^ Potential mechanisms for the observed drastic increase of sensor response to both analytes due to Pd embedding are assessed revealing the role of Pd near the SnO_2_ surface and Pd/PdOx domains into its bulk.

As our focus is on understanding the partitioning of Pd between the surface and interior of SnO_2_ and the subsequent impact on gas sensing, we benchmarked the sensitivity, the most basic property of gas sensors, at a commonly employed temperature, humidity and concentration of acetone and CO. Other sensor characteristics such as selectivity, stability and response and recovery times have not been investigated as they largely follow those of pure and Pd-containing SnO_2_ sensors. The effect of sensing temperature, humidity and analyte concentration on the performance of SnO_2_-based sensors has been documented extensively for acetone^13,15–18^ and CO^11,16,19–22^ as summarized in Table S1 in the ESI.†

## Experimental

### Synthesis of sensing nanoparticles

Pure and Pd-doped SnO_2_ particles were prepared by FSP.^13^ A precursor solution of tin(ii)-ethylhexanoate (Aldrich, purity >92.5%) and palladium(ii)-acetylacetonate (Aldrich, purity ≥99%) in xylene (Aldrich, purity >96%) with a total metal ion (or precursor solution) concentration, *C*, of 0.1, 0.5, 1.0 or 1.5 M was used. Palladium was added at the concentration of 0, 0.1, 0.2, 0.5, 1 and 3 mol% of total metal content. This solution was fed to the capillary of a concentric spray nozzle at 1, 5 and 9 ml min^−1^ by a syringe pump and dispersed by an annular oxygen flow of 2, 5 or 8 l min^−1^. A premixed methane/oxygen (1.3/3.2 l min^−1^) flame ignited and supported the spray flame. A circular sheath oxygen flow of 5 l min^−1^ was used around that flame to assure complete combustion of the precursor. The FSP inlet conditions for all experiments are listed in Table S2 in the ESI.†

Using a vacuum pump, the powder was collected (Busch, Seco SV 1025C) on a glass-fiber filter (GF6 Albet-Hahnemuehle, 257 mm diameter) 50 cm above the burner. Product powders were scraped off the filter surface with a spatula and sieved with 0.25 mm stainless steel sieve to remove filter fragments. A portion of all powders was annealed in air at 500 °C for 5 hours (Carbolite Gero, Gero 30–3000 °C). Both as-prepared and annealed powders were kept for further characterization.

### Removal of surface Pd

First, 150 mg of annealed powder were placed in a quartz reactor tube and secured with glass wool on both ends. The powder was then reduced by passing 100 ml min^−1^ of 5% H_2_ in Ar for 30 min at 150 °C. Then 100 mg of such reduced powder was placed inside a round bottom flask where 85 ml of ultrapure water (Merck Millipore) and 15 ml of nitric acid (65% HNO_3_ in water, purity ≥99.99%) were added. Then the flask was heated up to 60 °C in an oil bath and stirred under reflux for 4 h at 500 rpm to dissolve the palladium on the surface of SnO_2_ particles.^13^ Then the flask contents were transferred to falcon tubes and the particles were separated from solution by centrifugation (7200 of relative centrifugal force for 10 min), washed thrice with ultrapure water and dried in a vacuum oven (SalvisLAB, Vacucenter) at 50 °C and 50 mbar for 12 h. The resulting leached solution was analyzed with inductively coupled plasma-optical emission spectroscopy (ICP-OES, Agilent 5110) to determine the amount of leached Pd. The ICP-OES was calibrated using standards for Sn and Pd in HNO_3_ solutions (inorganic ventures). The surface palladium fraction was defined as the ratio of the measured amount of leached palladium and the nominal Pd content in the powder. The SnO_2_ powders with Pd content 0, 0.1, 0.3 and 0.5 mol% were completely digested by adding 3 ml concentrated HNO_3_ (69%) and 500 μL HF (40%) and heating to 240 °C for 10 min in a pressurized microwave (UltraClave, MLS GmbH). Digestion was performed both on annealed and leached powders to determine the total and embedded Pd content. After digestion, samples were filled up to 50 ml with ultrapure water. The concentrations of Sn and Pd ions then were measured by ICP-OES (Agilent 5110) as the above leached solutions.

### Particle characterization

Particle phase composition and crystal size, *d*, were obtained by X-ray diffraction (XRD, Bruker D2 Phaser) at 40 kV and 40 mA at 2*θ* (Cu Kα) of 20–70° with a step size of 0.1° and scanning speed of 0.0972° s^−1^. The patterns and crystal size were determined by Rietveld fundamental parameter refinement with the Topaz 4.2 software (Bruker) using the characteristic parameters of cassiterite SnO_2_ (PDF 41-1445) and Pd (PDF 01-1310) and PdO (PDF 43-1024).

The specific surface area (SSA) and surface equivalent particle diameter (*d*_BET_) of the powders were measured by 5-point N_2_ adsorption at 77 K (Micromeritics Tristar II Plus) using the Brunauer–Emmett–Teller method. Powder samples were degassed under N_2_ at 150 °C for 1.5 h prior the adsorption measurement. The *d*_BET_ was calculated using the densities of SnO_2_ (6.95 g cm^−3^) and PdO (8.3 g cm^−3^).

Specimens for transmission electron microscopy were prepared by dispersing a spatula tip of dry powder in 500 μL of high purity ethanol (>99.9%, LiChrosolv, Supelco) in an agate mortar. A volume of 10 μL of supernatant from the mortar was then drawn through a lacey carbon TEM grid (EM resolutions) using a tissue. Then, the grid was washed thrice with ultrapure water and air dried. High-angle annular dark-field scanning transmission electron microscopy (HAADF-STEM) images and energy-dispersive X-ray (EDXS) elemental maps were then obtained from these specimens using a FEI Talos (F200X, Super-X EDS, 4 detector configuration, FEI, Hillsboro, OR, USA).

X-ray Photoelectron Spectroscopy (XPS) measurements were conducted on samples of as-prepared, annealed and leached powders containing 1 and 3 mol% of Pd, as for that with 0.2 mol% Pd, the signal to noise ratio was too low for analysis. Powders were compressed into separate indium foil pieces and subjected to analysis using a Quantum 2000 XPS system (Physical Electronics). Detailed descriptions of these measurements can be found in the ESI.† Literature binding energies for relevant Pd species along with respective average, min/max and standard deviation values that were used to constrain the curve fitting model are shown in Table S3, ESI.†

### Sensor assembly and measurements

Powders were mixed with 1,2-propanediol (Sigma-Aldrich, purity >> 99%) to form viscous and homogeneous pastes. The pastes were doctor bladed^13^ with a razor on Al_2_O_3_ sensor substrates (Electronic Design Center, Case Western Reserve University, Electrode type #103) having two interdigitated Pt electrodes (sputtered, 350 μm width and spacing) on the front and a Pt heater on the back. The sides of the substrates were secured with 15 μm thick aluminum foil to control the thickness of the spread film. The sensors were dried at room temperature for 4 hours, followed by 16 hours at 80 °C. Finally, the sensors were annealed for 30 minutes at 300 °C with 10 °C min^−1^ ramping to improve mechanical stability of the film and remove any remains of organics used during paste preparation. An illustration depicting the sensor, outlining the measurement principle of film resistance (*R*) and demonstrating the doctor-blading procedure can be found in Fig. S1 and S2, respectively, within the ESI.†

The sensor was placed in a closed chamber and heated to 350 °C by DC through its substrate Pt heater, adjusted by linear regression for temperature and the resistance was determined individually for each sensor using thermocamera (Fluke, Ti110). A synthetic air stream of 1 l min^−1^ and 50% relative humidity (RH) was continuously passed through the chamber with the sensor until a stable baseline resistance (*R*_air_) was achieved. The humidity was tuned by mixing a stream of dry synthetic air with an equal stream of air saturated with water vapor in an inline water-filled bubbler. To measure sensor response to analytes, acetone or carbon monoxide was admixed to the air stream from calibrated gas standards in an appropriate amount to result in a concentration of 1 ppm. The sensor exposure to analyte lasted for, at least, 10 minutes to ensure that resistance has reached a final value (*R*_analyte_). The interval between two consecutive pulses was, at least, 20 minutes to ensure the resistance returned to the baseline. The sensor response (*S*) is:1
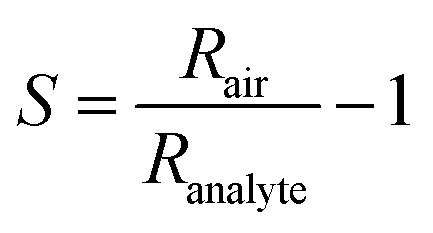
as SnO_2_ is an n-type semiconductor and both acetone and carbon monoxide are reducing gases.^23^ The sensor response and recovery times were defined as the time needed for the sensor resistance to reach 90% of the final change upon analyte exposure or 90% of recovery after the dosing of the analyte has been discontinued.

## Results and discussion

### SnO_2_ crystal size control

**Fig. 1:**
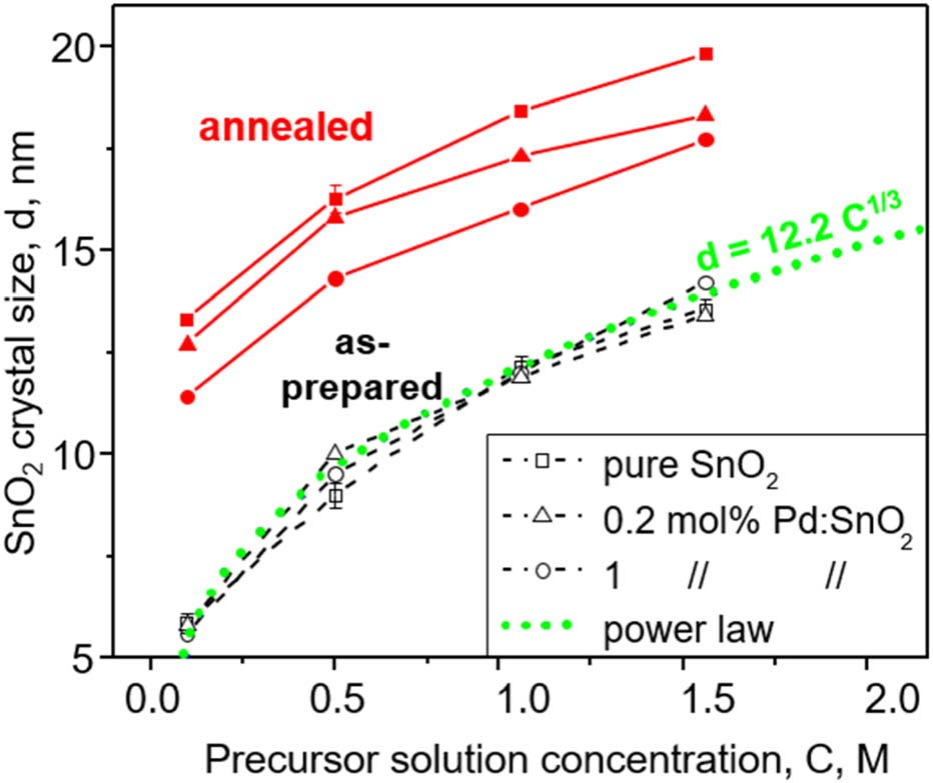
Crystal size of as-prepared (black, open symbols) and annealed (red, filled symbols) pure (squares) and doped SnO_2_ with 0.2 (triangles) and 1 mol% Pd (circles) made at FSP precursor solution flowrate, P (ml min^−1^), to dispersion O_2_ flowrate, *D* (l min^−1^), of 5/5 as a function of FSP precursor solution concentration (*C*).

Fig. 1 shows the as-prepared (open symbols) and annealed (filled symbols) SnO_2_ crystal size, *d*, as a function of the FSP precursor solution concentration, *C*, for pure (squares) and doped SnO_2_ with 0.2 (triangles) and 1 mol% Pd (circles) made with the ratio of precursor solution flowrate (*P*, ml min^−1^) to dispersion O_2_ flowrate (*D*, l min^−1^), *P*/*D* = 5/5. Increasing C enlarges the SnO_2_ crystals. In contrast, the Pd content had no effect on the as-prepared SnO_2_ crystals that increased from 5.6–5.8 nm to 13.4–14.2 nm with C increasing from 0.1 to 1.5 M. In contrast, increasing the Pd content hindered the growth of annealed SnO_2_ by the solute drag effect^24^ as shown also in Fig. S3 in the ESI† in agreement with Pineau *et al.*^13^ Given that the particle size as determined by N_2_ adsorption (Fig. S3, ESI,† squares) was close to the crystal size (Fig. S3, ESI,† circles) at all Pd contents for both as-prepared and annealed particles, monocrystalline SnO_2_ particles were made here.

Most interestingly, the size of all as-prepared particles followed a power law (Fig. 1, dotted line) with *C*, *d* = 12.2C^1/3^, indicating that particle size directly relates to precursor solution concentration, *C*, and consequently to formation from single droplets by evaporation-precipitation.^25^ Most likely, such droplets were formed by microexplosions.^26^ So SnO_2_ formation takes place largely by droplet-to-particle conversion at the highly oxidic conditions of spray combustion.

Increasing the ratio of FSP precursor solution to dispersion O_2_ flowrate, *P*/*D*, increases the precursor aerosol concentration and flame temperature (Fig. S4 in the ESI†) that increase SnO_2_ crystal size (Fig. S5, ESI†) as seen in Fig. 1 with increasing *C*. However, the increased flame temperature at high *P*/*D* results in slightly larger SnO_2_ crystals, *i.e.* 12 nm at *P*/*D* = 5/5 (Fig. 1) and 15 nm at *P*/*D* = 5/2 for pure SnO_2_ (Fig. S5, ESI†). It should be noted that the SnO_2_ crystal size did not change much due to reduction with H_2_, leaching and subsequent washing and drying.

### Palladium distribution and oxidation state

Metallic Pd can be leached from the SnO_2_ surface as shown for photodeposited Pd onto SnO_2_, where more than 99% of the nominal Pd content was removed.^13^ For flame-made particles, the question arises regarding the partition of Pd between the particle's surface and interior, as well as its oxidation state. All annealed Pd/SnO_2_ particles were leached with HNO_3_ to remove Pd from the SnO_2_ surface and determine the Pd surface fraction from the Pd ion concentration in the leachate and subsequently the embedded fraction of the total Pd content. To confirm results from HNO_3_ leaching, Pd/SnO_2_ powders with Pd content ranging from 0.1 to 0.5% of Pd were completely digested using HF. Digestion was performed before and after leaching to determine the total Pd and the embedded Pd content. Solutions remaining after HF digestion were diluted and the concentrations of Sn and Pd ions were measured by ICP-OES.

Juxtaposition of the results of HNO_3_ leaching and HF digestion experiments is shown in Fig. S6 in the ESI.† The sum of Pd determined in the leachates and that in the residual material after leaching was close to 100% of the nominal Pd content in the SnO_2_ powder for all Pd-contents.

**Fig. 2:**
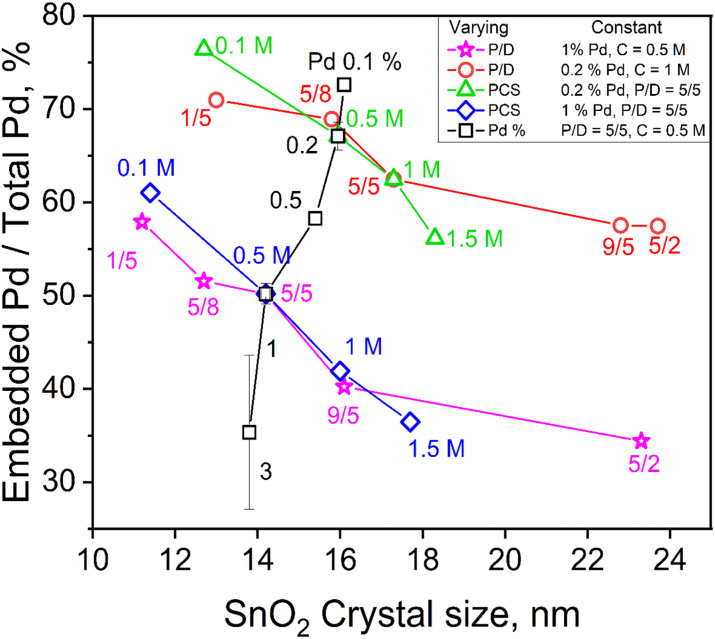
The embedded Pd fraction as a function of SnO_2_ crystal size in the annealed powders made by varying one of the FSP process variables, *P*/*D* (stars and circles), *C* (diamonds and triangles) or Pd content (squares), while keeping the other two constant. The actual embedded Pd content (mol%) as a function crystal size is provided in Fig. S7 in the ESI.†

To investigate the impact of FSP process conditions on the embedded content of Pd, one FSP process variable (precursor concentration, *C*, precursor solution/dispersion O_2_ flowrate ratio, *P*/*D*, or Pd content, Pd%) was varied while the other two were kept constant. For example, the fraction of embedded Pd into SnO_2_ from total (nominal) Pd contents of 0.1, 0.2, 0.5, 1 and 3 mol% produced at *C* = 0.5 M and *P*/*D* = 5/5 is shown as a function of SnO_2_ crystal size in Fig. 2 (squares). Increasing the total Pd content, drastically decreases the embedded Pd fraction from 73 to 35% as the actual amount of embedded Pd increases into SnO_2_ since the total Pd content in SnO_2_ increases (Fig. S7 in the ESI†).

Increasing the *C* from 0.1 to 1.5 (Fig. 2, triangles) and *P*/*D* from 1/5 to 5/2 (circles) at 0.2 mol% nominal Pd content hardly altered the mole fraction of embedded Pd in SnO_2_ (Fig. S7 in the ESI†), even though the embedded fraction decreased from 76 to 56% of the nominal value. The same increase of C (Fig. 2, diamonds) and *P*/*D* (stars) at higher Pd content, 1 mol%, decreased the embedded fraction of Pd from 60 to 35% as both FSP variables increase SnO_2_ crystal size while inversely affect the flame temperature (Fig. S4 in the ESI†) indicating its limited significance at the employed conditions. As SnO_2_ particles are formed by droplet-to-powder conversion (Fig. 1), the precursor solution concentration, *C*, largely determines their size. Precursor solutions of increasing *C* and/or *P*/*D* increase the metal ion concentration and form larger SnO_2_ crystals (Fig. 1 and S7 in the ESI†). As particle formation takes place within microdroplets^26^ (Fig. 1), Pd and Sn are well mixed there and embedding of Pd species into the SnO_2_ matrix takes place.

**Fig. 3:**
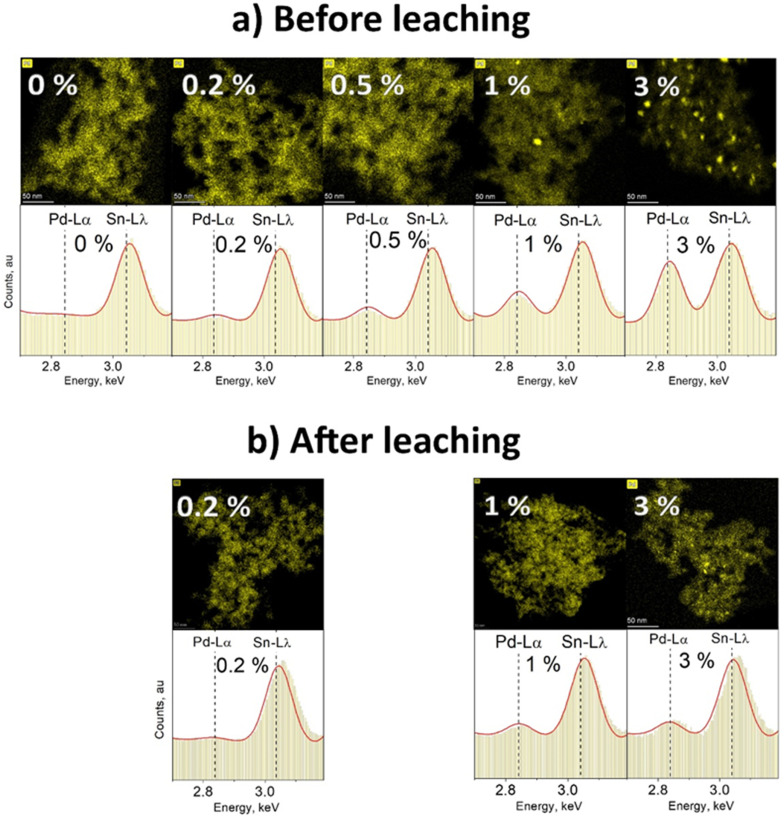
(a) Top panel: Pd elemental maps of annealed SnO_2_ doped with Pd contents of 0, 0.2, 0.5, 1 & 3 mol% and made at *C* = 0.5 M, *P*/*D* = 5/5. Bottom panel: EDXS spectra corresponding to the image area above. All images were recorded at the same imaging conditions (450 kx magnification, beam current of 8 nA, 10 frames at 50 μs dwell time). As the content of Pd increases, more and larger Pd-rich (bright) spots are visible. (b) Top panel: Pd elemental maps of SnO_2_ containing originally 0.2, 1 & 3 mol% Pd after undergoing leaching with HNO_3_. Only in the 3 mol% sample some Pd-rich spots are visible. The bottom panel: EDXS spectra corresponding to the image area above. Clearly, the amount of Pd in relation to Sn has decreased compared to these samples before leaching and corresponds to the embedded Pd into SnO_2_.

To visualize the change in content and dispersion of Pd in and/or on the particles at different Pd doping levels, STEM-EDXS mapping was used. Fig. 3 presents EDXS elemental maps of annealed SnO_2_ doped with various amounts of Pd (a) before and (b) after leaching with nitric acid. The change of particle Pd content can be observed on the basis of the respective image EDXS spectrum shown in the lower panel of Fig. 3a. As the Pd content increases from 0 to 3%, the relative height of the characteristic Pd-L_α_ peak at 2.839 keV increases as well. Even though there is no Pd peak in the EDXS spectrum of pure SnO_2_ (0 mol% Pd, Fig. 3a), it still appears on the elemental map above. The Pd map in this case is only based on unspecific *Bremsstrahlung* X-ray counts, generated once material is within the path of the electron beam. The exact spectrum for the different Pd map areas for pure SnO_2_ in Fig. S8cd in the ESI† proves that there is no detectable Pd in this sample. Before leaching, pure SnO_2_ (0% Pd in Fig. 3a) showed the characteristic Sn L_λ_ peak at 3.045 keV and was free from Pd L_α_ X-ray counts at 2.839 keV. Using a high beam current of 8 nA, Pd down to 0.2 mol% was detected at 2.839 keV of the EDX spectrum. At the lowest Pd-contents of 0.2 & 0.5 mol%, virtually no Pd clusters were visible while still a distinct Pd peak could be seen at 2.839 keV. This suggests that Pd is distributed inside and/or on the surface of SnO_2_ – atomically dispersed or in Pd clusters close to or below ≈2 nm in diameter (LOD).

In the elemental map for SnO_2_ containing 1 mol% Pd before leaching (Fig. 3a), single Pd clusters larger than 2 nm can be seen. Also for 3 mol% Pd before leaching (Fig. 3a) even more Pd-rich spots with a diameter from a few to a dozen nm can be observed. These observations are in line with Koziej *et al.* for impregnated^27^ Pd and Gschwend *et al.* for photodeposited^11^ Pd onto SnO_2_. Using K-edge XANES & EXAFS, for example, Koziej *et al.*^27^ found that at low Pd-contents, *e.g.* 0.2 mol%, Pd is finely dispersed and does not form any clusters which only become apparent at high Pd concentrations (3 mol%).

After leaching, no Pd clusters were detected for 0.2 mol% Pd (as expected) and hardly any for 1 mol% Pd (Fig. 3b). When comparing the latter to its initial sample, we go from some to virtually no clusters, indicating that most of the Pd clusters were actually on the SnO_2_ particle surface. However, when 3 mol% Pd/SnO_2_ was leached, still some Pd clusters were visible. Their number and average size were smaller than before leaching, but still single clusters up to 10 nm in diameter were present. Such clusters could be embedded in SnO_2_ which would protect them from the HNO_3_ leaching. Another possibility is that such visible Pd-rich regions are not clusters, but only superficially Pd-enriched SnO_2_ areas that were in contact with larger Pd clusters prior to their leaching. Such enrichment might be caused by annealing-induced solid-state diffusion of Pd into SnO_2_ and/or solid-state reaction^13^ forming PdSnO_*x*._ This is supported by the fact that the fraction of leachable Pd decreases by 20–40% after annealing.^13^

Clearly very small amounts of Pd can be incorporated into SnO_2_ by FSP that could form solid solutions. Such atomic Pd doping should occur only to a limited extent as the SnO_2_ diffraction patterns were not affected by Pd at all Pd contents employed here (Fig. S9 and S10 in the ESI†). However, with increased Pd loading, Pd forms a separate phase (*i.e.* Pd/PdO_*x*_) that cannot be detected by XRD. This phase resulted in domains and clusters into and onto the SnO_2_ proportional in size and number to the nominal Pd content as shown in Fig. 3a. Larger clusters are not easily embedded in SnO_2._ As a result, more Pd would be exposed to leaching, decreasing the embedded Pd fraction at higher nominal Pd loadings (*i.e.* only 35% is embedded at the 3 mol% nominal Pd loading, Fig. 2).

To more closely investigate the oxidation states of Pd, X-ray Photoelectron Spectroscopy (XPS) analysis was performed (Fig. S11†). Due to the limited depth of analysis (typically ≈1–5 nm),^28,29^ this technique provides valuable information about the surface and near-surface regions rather than the bulk of the SnO_2_ powder. Detailed spectra with deconvolution into individual oxidation states are presented in the ESI, Fig. S12 and S13.† They revealed the dominating presence of oxidized forms (PdO_*x*_) of palladium as well as metallic Pd^0^ in all the as-prepared and annealed (unleached and leached) samples (Fig. S14 in the ESI†).

The measurements showed PdO_*x*_ : Pd^0^ ratios of ≈1.1 and ≈0.7 for particles containing 1 and 3 mol% of Pd, respectively, for both as-prepared and annealed samples (Fig. S15, ESI†). These are consistent with Deligiannakis *et al.*^30^ who found a PdO_*x*_ : Pd^0^ of 0.8 for FSP-made Pd/TiO_2_. As the boiling point of metallic palladium is 2963 °C and the decomposition temperature of palladium oxide is around 800 °C,^31^ palladium clusters formed in high temperatures regions of the flame will possess a metallic character. However, as these clusters reach temperatures of around 700 °C away from the flame, they undergo oxidation^32^ but only at the surface layer which has access to oxygen. The Pd clusters core and portion incorporated into SnO_2_, lacking access to oxygen, would remain in their metallic form as confirmed by the XPS spectra.

After leaching however, the PdO_*x*_ : Pd^0^ ratio increased dramatically to 2.2 and 2.8 for 1 mol% and 3 mol% initial Pd, respectively (Fig. S15, ESI†) suggesting a lower content in metallic Pd after leaching. In this case, it was possible to remove all Pd from the surface, suggesting surface only Pd can be fully reduced to Pd^0^ after the reduction process, as only Pd^0^ is soluble in HNO_3_.^11^ However, in this study, Pd was introduced already in the flame and thus mixed into the entirety (inside and outside) of the SnO_2_ particles. Therefore, here, it is possible that – as suggested from XPS – a portion of metallic Pd can remain shielded beneath a protective PdO_*x*_/SnO_2_ surface.

In a first line, the XPS results demonstrate that the particle surface is dominated by oxidized Pd species. However, they also suggest the presence of metallic Pd, most likely near the surface. Whether this is true and valid also for deeper layers of the particles warrants further investigation by complementary and bulk analysis techniques such as X-ray absorption spectroscopy.

### Sensing performance

**Fig. 4:**
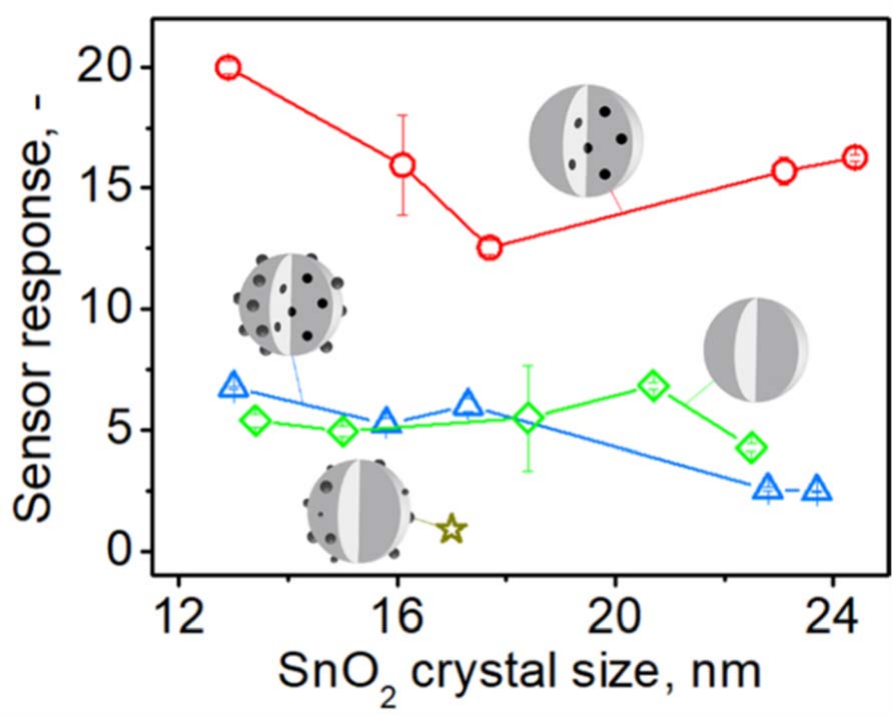
The response to 1 ppm of acetone at 350 °C in 50% RH by sensors made with pure (green diamonds) and 0.2 mol% Pd-containing SnO_2_ particles before (blue triangles) and after HNO_3_ solution leaching and removal of surface Pd (red circles) made at various FSP precursor solution to dispersion O_2_ flowrate ratio (starting from the left *P*/*D* = 1/5, 5/8, 5/5, 9/5, 5/2) and *C* = 1 M as a function of SnO_2_ crystal size. The star shows the response of a SnO_2_ sensor that contained 0.2 mol% photodeposited Pd.^11^ Schematics of sensing particles depict with gray the SnO_2_ and black the Pd/PdO_*x*_ atoms, clusters or domains.

To investigate the impact of embedded Pd on the sensing of organics (acetone) and inorganics (CO), the response of SnO_2_ sensors containing (a) embedded and surface Pd, (b) only embedded and (c) only surface Pd was compared. Fig. 4 shows the sensor response to 1 ppm of acetone at 350 °C in 50% RH by pure (green diamonds) and 0.2 mol% Pd-doped SnO_2_ (an optimal Pd content^33,34^ for gas sensors) as a function of SnO_2_ crystal size made at various *P*/*D* at *C* = 1 M (circles in Fig. 2).

For all sensors, increasing the *P*/*D* and the resulting SnO_2_ crystal size from 13 to 24 nm (Fig. S5 in the ESI†) hardly affects their response consistent with Güntner *et al.*^12^ Pure SnO_2_ sensors had a stronger response (green diamonds) than those containing only surface Pd (gold star)^11^ but comparable to those having both surface and embedded Pd (blue triangles). However, when surface Pd was removed from the SnO_2_ particles, their response (red circles) increases by 2 to 6 times over pure (diamonds) and Pd-containing SnO_2_ (triangles) that had 50% more Pd, regardless of SnO_2_ crystal size.

These observations are attributed to the detrimental effect of surface Pd (Fig. 4, gold star) and the enhancement of sensing provided by embedded Pd, as depicted in Fig. 4 (red circles), which is fully manifested only after removal of surface Pd. If the sensing reaction takes place mostly locally at the surface of noble metal clusters, no electron transfer to the conduction band of SnO_2_ will occur and thus no change in the conductivity of the sensing film.^35^ Therefore, it is commonly observed that sensors exhibit a maximum response when there is only a small amount of dopant present, as sensitivity decreases with increasing dopant content. For SnO_2_ containing both surface and internal palladium (Fig. 4, triangles), it is plausible that their effects mutually negate each other.

When the same sensors were tested towards 1 ppm of CO at the same conditions (Fig. S16 in the ESI†), the response for all sensors again was hardly affected by SnO_2_ crystal size^12^ from 13 to 23 nm. The response of the pure SnO_2_ sensors (green diamonds) is lower or comparable to that containing both fractions of Pd (blue triangles). Again, when the surface Pd is removed, the sensor response (red circles) increases 2–6 times over that of pure SnO_2_ (green diamonds) and that containing both surface and embedded Pd (blue triangles).

**Fig. 5:**
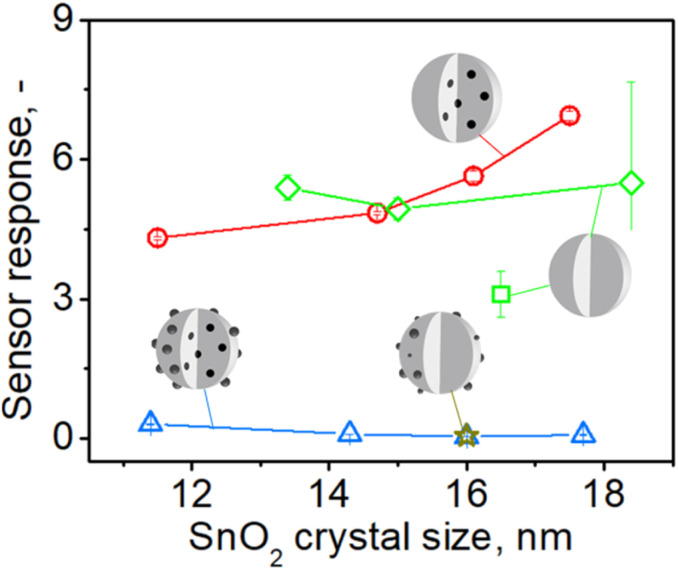
The response to 1 ppm of acetone at 350 °C in 50% RH by pure (green square,^13^ diamonds – responses from Fig. 4) and 1 mol% Pd-containing SnO_2_ sensors before (blue triangles) and after leaching and removal of surface Pd (red circles) made at various FSP precursor solution concentrations (starting from the left *C* = 0.1, 0.5, 1, 1.5 M) and i/*D* = 5/5 as a function of SnO_2_ crystal size. The star shows the response of a sensor that contained 1 mol% photodeposited Pd onto SnO_2_ particles by FSP at *C* = 0.5 M and same *P*/*D*.^11^

To better understand this enhancement, sensors with high nominal palladium loading of 1% (or 0.6 to 0.4 mol% actual embedded Pd, Fig. S7 in the ESI†) made at increasing precursor solution concentrations *C* (and subsequently particle size from 11 to 18 nm, Fig. 1) were tested towards acetone at the same conditions as above (Fig. 5). Sensors containing only surface Pd (gold star) or both surface and embedded Pd (blue triangles) showed hardly any response to 1 ppm of acetone and well below that of pure SnO_2_ (green diamonds). When, however, surface Pd is removed, their response (red circles) becomes 14 to 95 times higher. This response is also higher or comparable to that of pure SnO_2_ (green square and diamonds). Nevertheless, please note that the best of these responses is 3–5 times lower than that of sensors containing 0.2 mol% Pd (Fig. 4). This is consistent with the literature showing that sensors with low Pd contents (0.1–0.2 mol%) exhibit higher response than those with higher Pd loadings (0.5–3 mol% Pd).^9,11,33,36^

When the above sensors were exposed to 1 ppm of CO (Fig. S17 in the ESI†), they gave similar results: hardly any response by those containing surface and embedded Pd (blue triangles), which is lower than that of pure SnO_2_ (green square and diamonds) and a much higher response by the sensor containing only embedded Pd (red circles) that reached a 125-fold amplification for the largest – 18 nm – SnO_2_ crystals (*C* = 1.5 M). The response of these sensors was also 2–3 times lower than that for 0.2 mol% Pd-doped sensors, similar to acetone (Fig. 4). Response times of about 10 seconds for pure SnO_2_ sensors were observed and a moderate increase to 20–40 seconds for those containing 0.2% Pd before its surface removal, in agreement with literature.^11^ Notably, leaching of surface Pd halved the response times to those before leaching. Recovery times of approximately 40–125 seconds were observed for pure SnO_2_ and 30 to 200 seconds for Pd-containing SnO_2_.

**Fig. 6:**
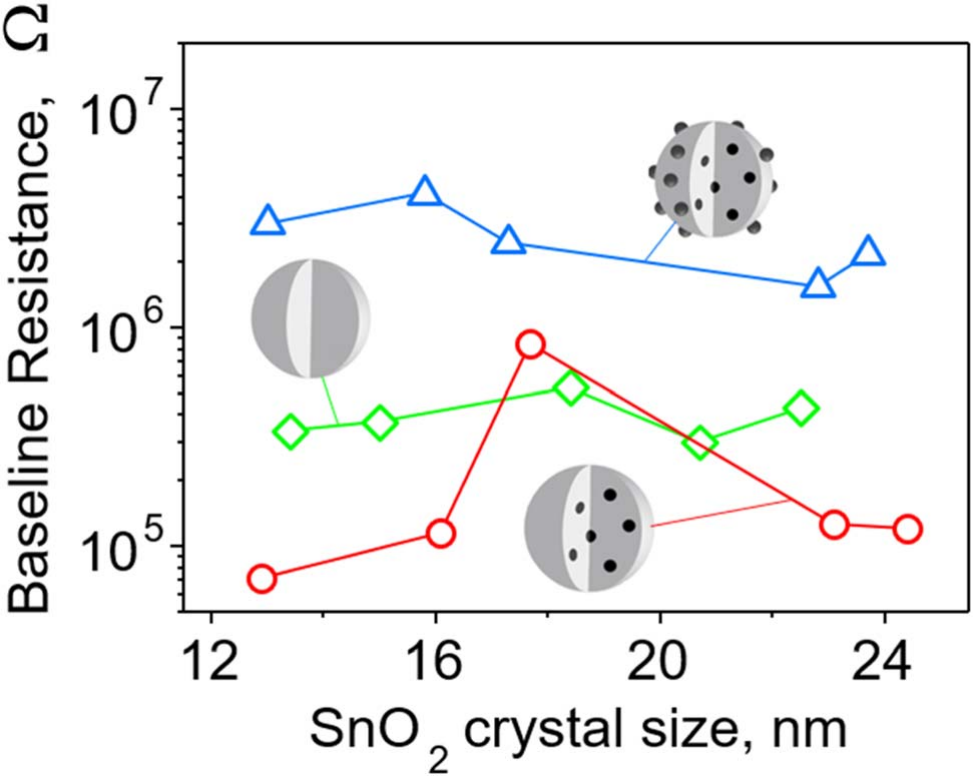
Baseline resistance of pure and 0.2 mol% Pd-doped SnO_2_ films as a function of SnO_2_ crystal size for particles made at various *P*/*D* (Fig. 4). Films of nanoparticles doped with Pd (blue triangles) both on the surface and in the bulk have the highest resistance – almost an order of magnitude higher than that of pure SnO_2_ film (green diamonds). Most films of nanoparticles with only embedded Pd (red circles) have resistance significantly lower than the other two SnO_2_ films.

To better understand the sensing mechanism of embedded Pd, the baseline of pure and Pd-containing SnO_2_ sensor was examined before and after leaching surface Pd from the latter ones (Fig. 6). The resistance of films containing 0.2 mol% Pd (surface and embedded, blue triangles) was about an order of magnitude higher than pure SnO_2_ (green squares) in agreement with Korotcenkov *et al.*^33^ This can be attributed to the presence of palladium oxide clusters on the surface. A similar picture holds also for sensors made with 1 mol% Pd (Fig. S18 in the ESI†). As mentioned before, there are two oxidation states of Pd, and thus both PdO and PdO_2_ can form surface clusters. The PdO_2_ is metallic while PdO, of which a significant fraction (up to 50%, Fig. S14, ESI,†) is found on/near the surface of the particles according to XPS measurements, is a p-type semiconductor.^37^ Band bending at binary and ternary heterojunctions with n-type SnO_2_ causes transfer of electrons and extension of the depletion layer and thus increases the film resistance.

When the surface Pd was removed, the film resistance fell even below that of pure SnO_2_ in all except one case (Fig. 6, red circles). This indicates that embedded Pd improves the conductive properties of the film possibly due to the shift of the Fermi level and/or the existence of nanoelectrodes.^38^ When Pd is dispersed at the atomic level within the crystalline lattice of SnO_2_, the electronic structure is likely to be changed through introduction of additional energy levels arising from defects in the crystalline structure introduced by dopant atoms.^39^ The atomically dispersed Pd within the SnO_2_ lattice has the potential to induce defects, such as oxygen vacancies, which are recognized as the primary source of n-type conductivity in^40^ SnO_2_ or Pd-related defects. Such defects can serve as charge carriers, increasing the material's conductivity and enhancing its response to analytes.^6^ The introduction of additional energy levels originating from defects can raise the position of the Fermi level to a higher energy, approaching the conduction band. This would facilitate the promotion of electrons to the conduction band, creating more free charge carriers (electrons) available for conduction and lowering the baseline resistance of the sensing film.

For SnO_2_ doped with 1 and 3 mol% Pd we also encounter Pd clusters with a diameter of a few nanometers. These are located within the SnO_2_ particle (Fig. 3b). It is presumed that the presence of embedded Pd or PdO domains can establish efficient conduction pathways within the film, thus minimizing its electrical resistance.^38^

Embedding Pd into the SnO_2_ bulk allows for synthesis of sensors characterized by both low resistance and high response, because there are no surface Pd clusters to compete chemically with SnO_2_. Embedded Pd clearly improves the transducing properties of the sensing film.

Alternatively, the observed increase in sensor response after leaching may be attributed to imparting specific surface properties to the material through the removal of external Pd clusters, which distinguish it from pure SnO_2_. These properties predominantly refer to increased surface catalytic activity. A similar technique to the leaching method employed in our study, aimed at removing surface-decorating metal clusters, was utilized by Yang and Flytzani-Stephanopoulos *et al.* to obtain single atoms of gold on CeO_2_ catalyst surfaces by treating them with a solution of NaCN.^41^ This treatment effectively eliminated the majority of metallic Au, leaving behind cationic Au atoms that formed strong bonds with CeO_2_ changing the surface properties of the catalyst. Similarly, in our investigation, the leaching selectively eliminates surface Pd clusters while allowing atomically dispersed Pd ions to remain within the SnO_2_ lattice, affecting surface activity similarly to single atom catalysts.^41–43^ As have been seen from XPS analysis (Fig. S14, ESI,†), the near-surface region of the particles still contained oxidized and metallic Pd. The Pd ions within the SnO_2_ lattice can provide initial adsorption sites for analyte molecules offering free valences and, by that, lowering their energy barrier of adsorption.^27^ Single Pd ions directly activate lattice oxygen which is not reactive at the pure SnO_2_ surface.^44,45^

## Conclusions

Tin oxide particles embedded with Pd were prepared by flame spray pyrolysis (FSP) of appropriate precursors followed by annealing and leaching metallic Pd from the surface of these particles with nitric acid. The SnO_2_ crystal size and the embedded fraction of Pd inside SnO_2_ were closely controlled by varying the FSP precursor solution concentration (*C*), total Pd content and the ratio of precursor solution (ml min^−1^) to dispersion oxygen (l min^−1^) flowrate (*P*/*D*). The response of sensors made with nominal Pd content of 0.2 and 1 mol% obtained at varying *C* and *P*/*D* was tested towards 1 ppm of acetone and carbon oxide (CO) at 350 °C and 50% relative humidity. The SnO_2_ crystal size increased with increasing *C* and *P*/*D*, while it decreased slightly with increasing Pd content. That way, SnO_2_ crystals in the range of 11–24 nm were obtained with the embedded palladium fraction ranging from about 20% to almost 76% of the nominal Pd content. As shown by Scanning Transmission Electron Microscopy (STEM) and Energy Dispersive X-ray Analysis (EDXS), at contents of 0.5 mol% and below, palladium was homogeneously dispersed within and/or on SnO_2_. Palladium domains and clusters smaller than 10 nm are visible at higher Pd contents, *e.g.* 1 and 3 mol%.

The SnO_2_ crystal size hardly affected the sensor response in the employed size range. The removal of surface palladium from 0.2 mol% Pd-doped SnO_2_ significantly enhanced (2–6 times) the sensor response to CO and acetone over that of pure SnO_2_ sensors and those containing both surface and embedded Pd and greatly exceeds (17–33 times) the response of SnO_2_ with the same nominal content (0.2 mol%) of photodeposited Pd. At high nominal Pd content, *i.e.* 1 mol%, the response of SnO_2_ sensors merely embedded with Pd is from one to two orders of magnitude higher than those containing both surface and embedded Pd.

This drastic enhancement of sensor response can be attributed to Pd atoms in the SnO_2_ lattice that may change charge carrier concentration and therefore conductance and responsiveness of the film. Also Pd atoms on/near the SnO_2_ particle surface can lower the adsorption energy of analytes while Pd/PdO clusters may act as nanoelectrodes enhancing the transducing properties of the sensing film.

## Author contributions

S. E. P. conceived the study. K. J. established and ran the experimental studies, evaluated analysis and proposed interpretation. C. M. P. K. assisted with the experiments. A. G. carried out the microscopic and XPS data analyses and proposed interpretations. K. J. and S. E. P. wrote the manuscript. All authors have given approval to the final version of the manuscript.

## Conflicts of interest

There are no conflicts to declare.

## Supplementary Material

NA-006-D3NA00558E-s001
