# Supplementary material for: Embedding Pd into SnO2 drastically enhances gas sensing
Source: Nanoscale Adv. 2024 Jan 31;6(4):1259–68. doi: 10.1039/d3na00558e (PMC10863718; doi:10.1039/d3na00558e)
Supplement: NA-006-D3NA00558E-s001 [file NA-006-D3NA00558E-s001.pdf]

Electronic Supplementary Material (ESI) for Nanoscale Advances.
This journal is © The Royal Society of Chemistry 2024

Electronic supplementary information:

Embedding Pd into SnO_2 drastically enhances gas sensing

Katarzyna Jabłczyńska,[a,b] Alexander Gogos,[c,d] Christian M.P. Kubsch [a] and Sotiris E. Pratsinis*[a]

[a]Particle Technology Laboratory, Institute of Energy and Process Engineering, Department of Mechanical and Process Engineering, ETH Zurich, CH-8092 Zurich, Switzerland

[b]Faculty of Chemical and Process Engineering, Warsaw University of Technology, 00-645 Warsaw, Poland

[c]Particles-Biology Interactions, Department of Materials Meet Life, Swiss Federal Laboratories for Materials Science and Technology (Empa), CH-9014 St. Gallen, Switzerland

[d] Nanoparticle Systems Engineering Laboratory, Institute of Energy and Process Engineering, Department of Mechanical and Process Engineering ETH Zurich, CH-8092 Zurich, Switzerland.

*S. E. Pratsinis pratsinis@ethz.ch, corresponding author

Submitted to

Nanoscale Advances

July 2023

and revised

December 2023

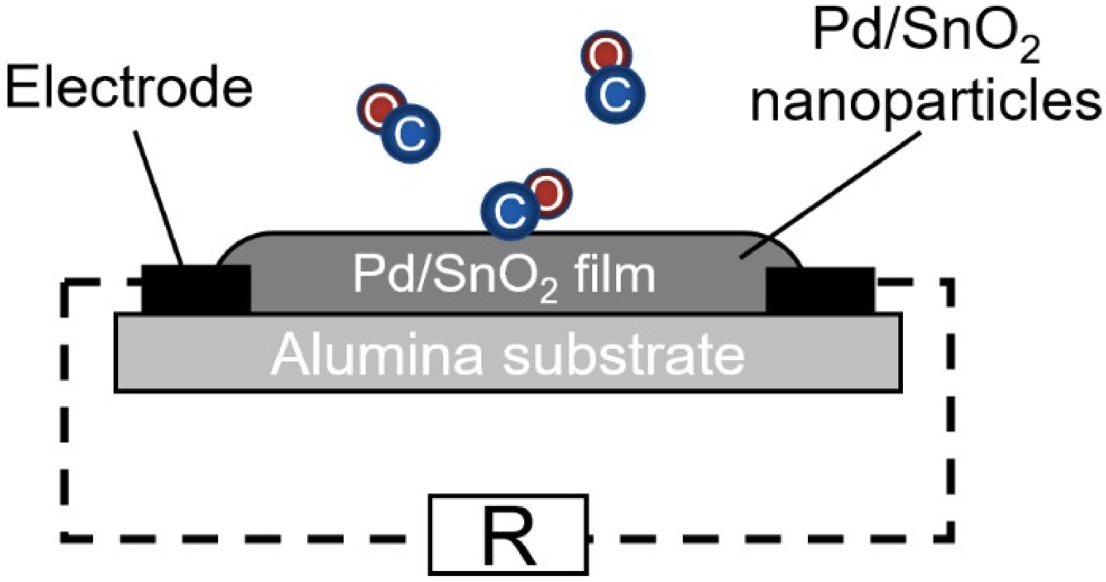

Fig. S1. The sensor with two platinum electrodes on an alumina substrate, connected via a nanoparticulate film. Alterations in electrical resistance between the electrodes are gauged upon exposure of the film to the analyte, e.g. CO or acetone vapor.

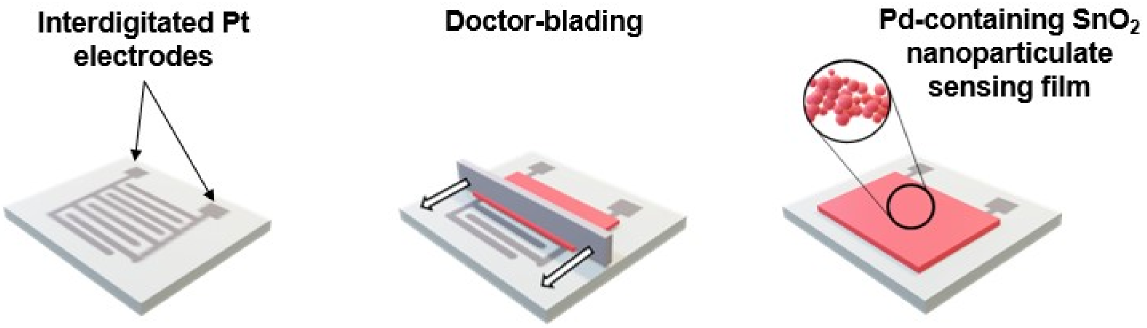

Fig. S2. The application of nanoparticle paste via the doctor-blading on the alumina sensor substrate featuring interdigitated platinum electrodes to create a sensing film.

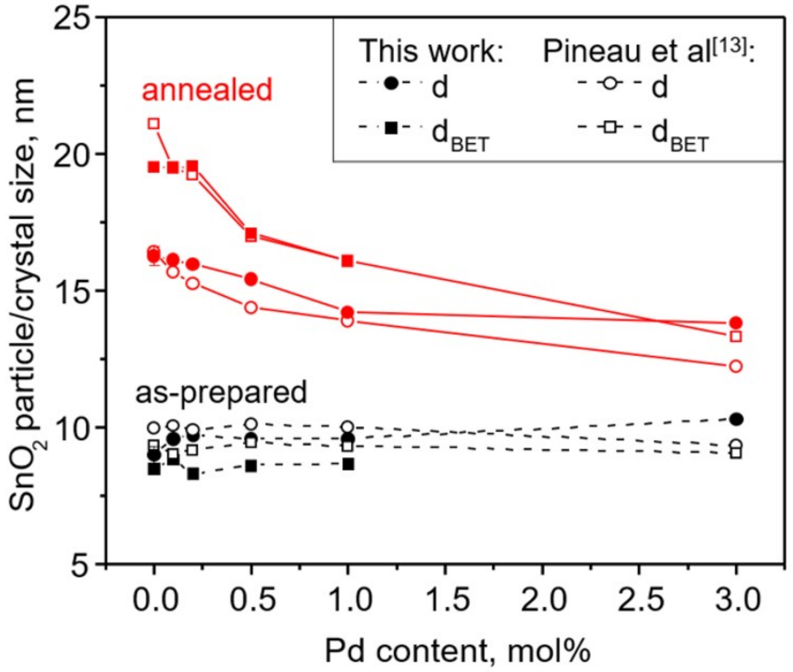

Fig. S3 Crystal size by XRD (circles) and particle size from N_2 adsorption (squares) of as-prepared (black broken lines) and annealed (500 °C in air for 5 h, red solid lines) SnO_2 particles/crystals that were made by flame spray pyrolysis (FSP) at 5 mL/min precursor solution flowrate, P, and 5 L/min dispersion O_2 flowrate, D, using a precursor solution with metal ions concentration of 0.5 M as a function of Pd molar content made here (filled symbols) in comparison to Pineau et al.[13] (open symbols).

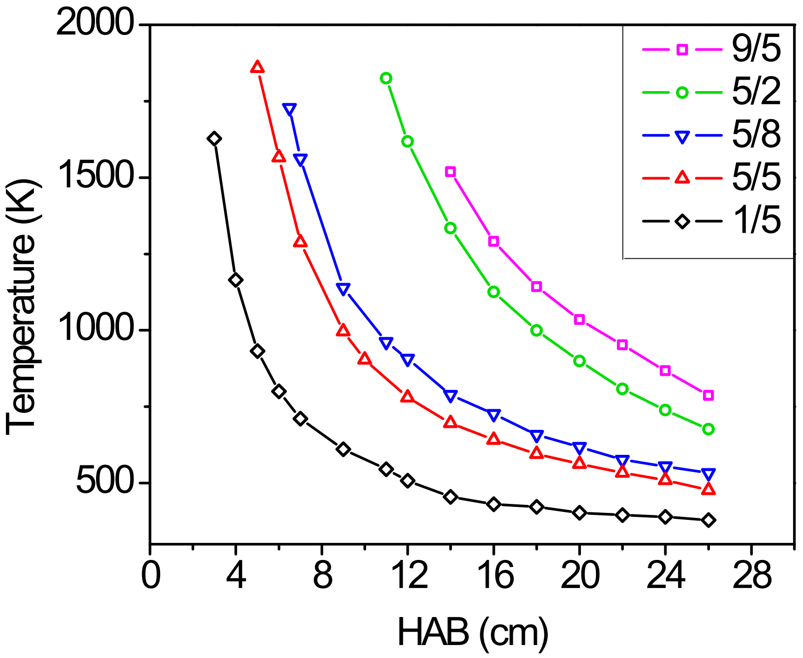

Fig. S4 Temperature at various heights above the burner (HAB) of xylene flames for selected P/D. The temperature above the burner was measured using an R-type thermocouple with 1 mm (nominal) bead diameter (Interotechno Friag AG) and was corrected for radiation. The thermocouple tip was placed in the central axis of the burner and kept at each HAB until the temperature reading was stabilized. The P/D influences both the concentration of metal ions and flame temperature as it supplies fuel for combustion.

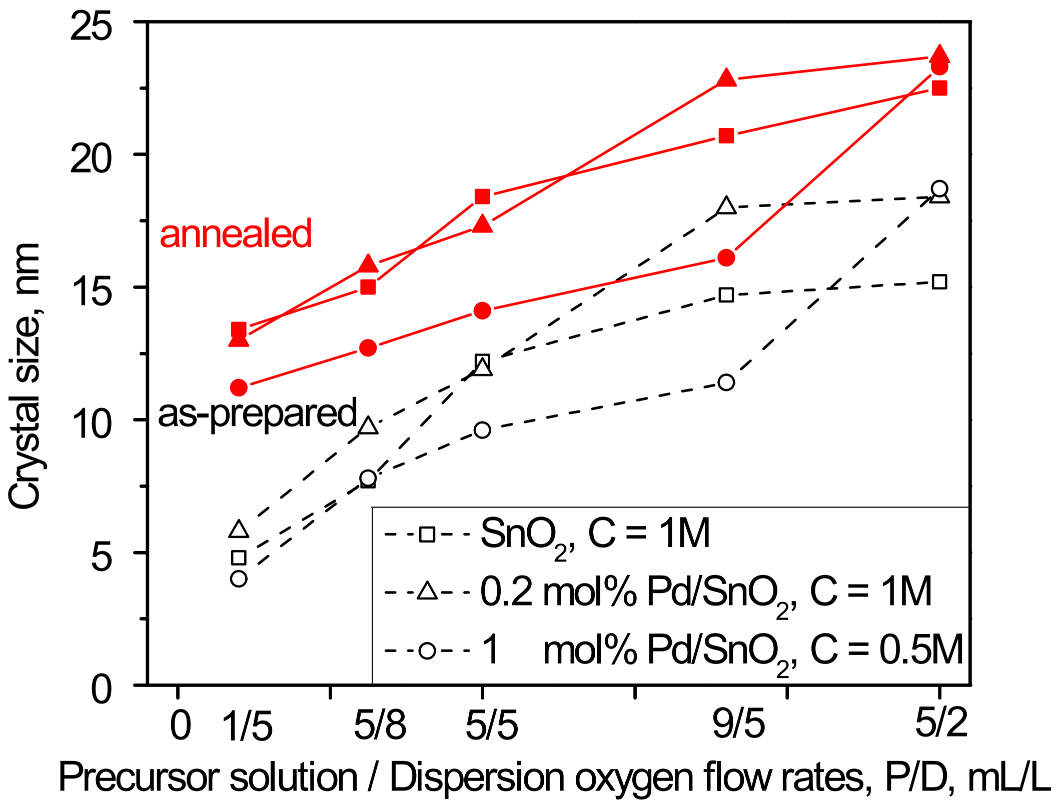

Fig. S5 Crystal size (as determined by XRD) of pure and 0.2 or 1 mol % Pd-doped SnO_2 as a function of precursor solution to oxygen flowrate ratio (P/D) at 1M or 0.5M, respectively, precursor solution concentration, C, before and after annealing @500 °C fo 5 h.

The figure shows the effect of precursor solution to dispersion oxygen flowrate ratio (mL/L), P/D, on SnO_2 crystal size of as-prepared and annealed pure and Pd-containing SnO_2 (0.2 and 1 mol%). The size of SnO_2 crystals produced at P/D = 5/5 is 12.1 before and 18.4 nm after annealing. These are in excellent agreement with Fig. 10 of Güntner et al.[12] where SnO_2 crystals were produced at exactly same conditions of size 12.2 and 18.8 nm, respectively. With increasing P/D, the crystal size of pure SnO_2 increases from 4.8 to 15.2 nm for the as-prepared powders and from 13.4 to 22.5 nm for the annealed ones. Similarly, increasing the P/D causes as-prepared Pd-doped SnO_2 to grow from 5.8 to 18.4 nm for 0.2 mol% Pd content and from 4 to 18.7 for 1 mol% Pd, while annealed crystals range from 13.0 to 23.7 nm and from 11.2 to 23.3 nm respectively. Note that a lower C was used for 1 mol% Pd.

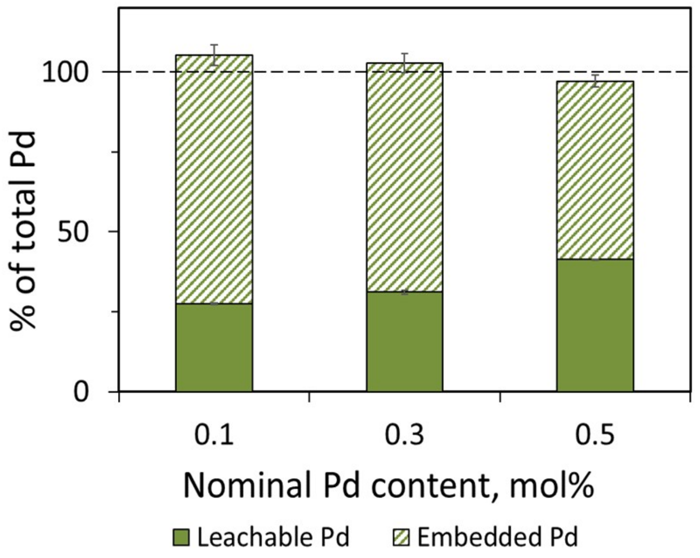

Fig. S6 Fraction of leachable (surface) and unleachable (embedded) fractions of Pd in Pd-containg SnO_2 particles at three nominal palladium contents.

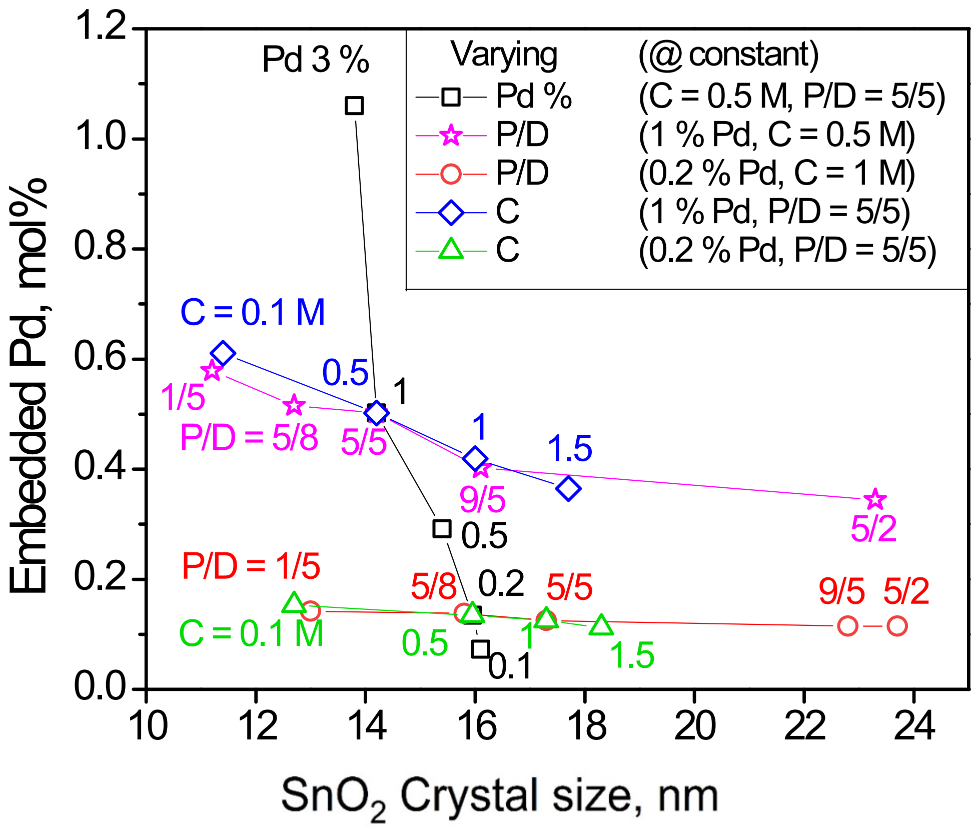

Fig. S7 The embedded Pd as a function of SnO_2 crystal size in the annealed powders made by varying one of the FSP process variables, P/D (stars, circles), C (diamonds, triangles) or Pd content (squares), while keeping the other two constant. Increasing the Pd content, at constant C and P/D (black squares) drastically increases the embedded Pd content even though the embedded fraction of the nominal Pd content decreases (Fig. 2) from 78% to 35%. Changing the Pd content hardly affected crystal size, as can be seen also in Fig. S3, with the size of as-prepared crystals to be constant (Fig. 1) even though SnO_2 crystal growth by annealing was inhibited by the presence of Pd.

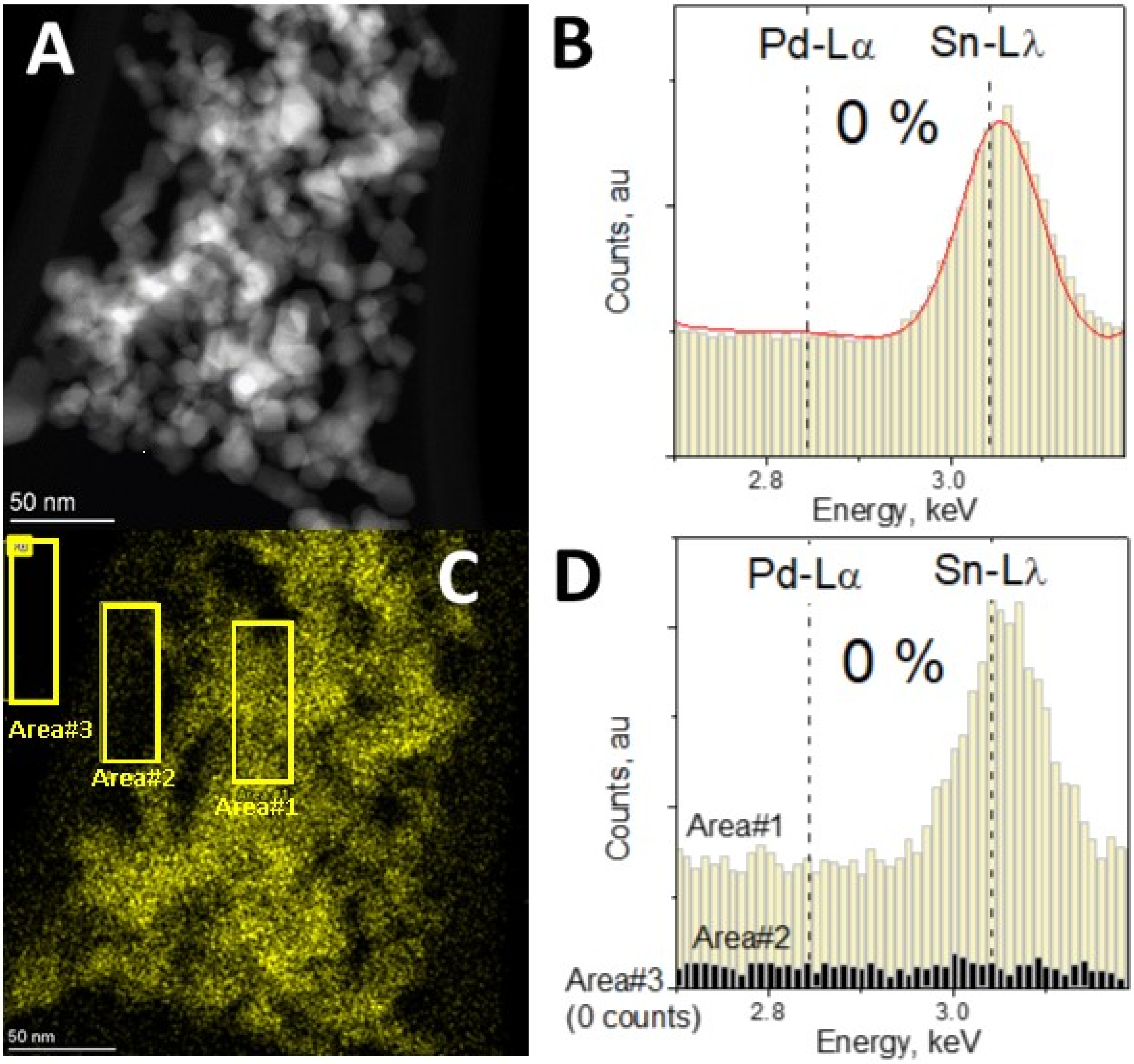

Fig. S8 A) HAADF micrograph of the pure SnO_2 base material and B) EDXS spectrum corresponding to the whole image area. C) Corresponding Pd map and (D) EDXS spectra obtained from the areas indicated in (C). These spectra demonstrate, that the Pd map in this case is only based on unspecific *Bremsstrahlung* X-ray counts, generated once material is within the path of the electron beam (Areas #2 & #3, Area#3 does not show any counts).

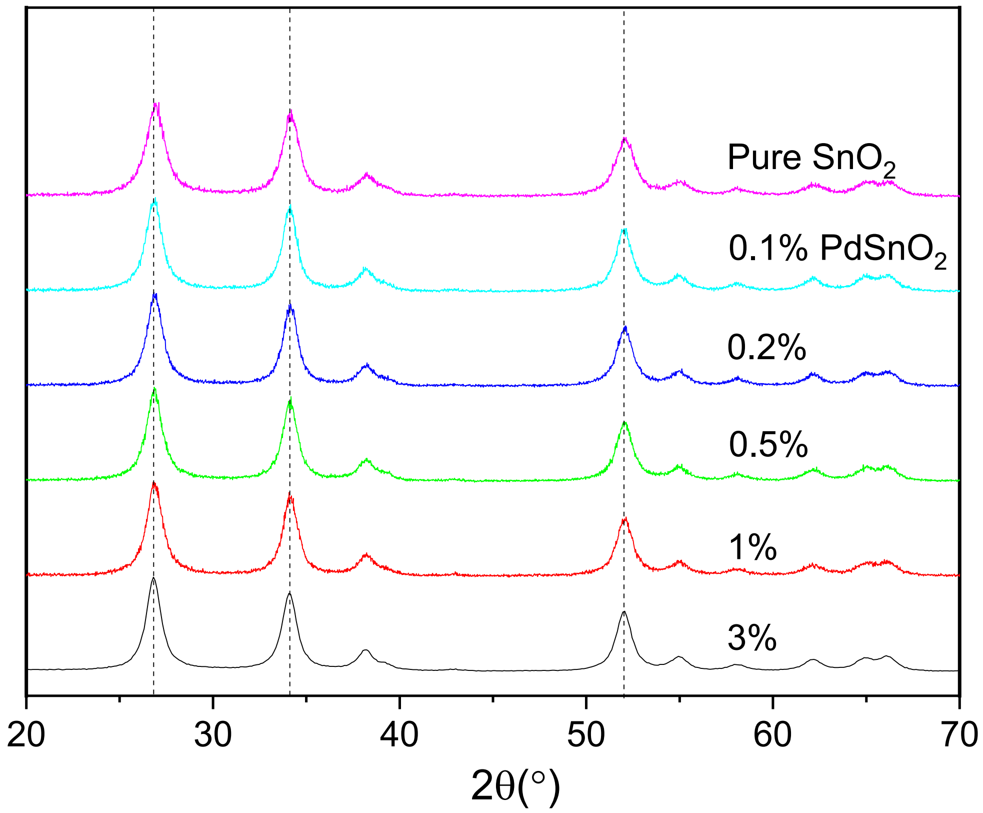

Fig. S9 XRD patterns of as-prepared SnO_2 doped with different Pd contents.

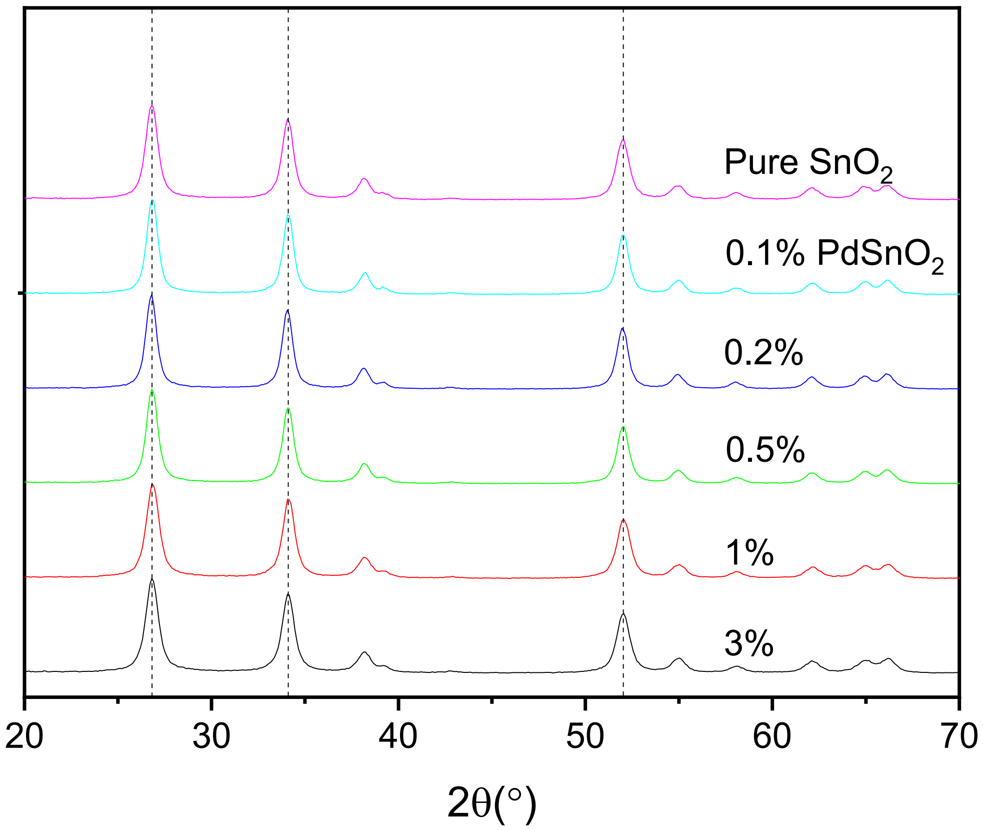

Fig. S10 XRD patterns of annealed SnO_2 doped with different Pd content.

Detailed X-ray Photoelectron Spectroscopy (XPS) experimental and results

For XPS analysis powders were pressed into individual pieces of Indium foil and measured on a Quantum 2000 XPS system (Physical Electronics). For each sample, individual survey spectra were collected. Afterwards, Pd 3d , Pd 3p and Sn 3d scans were collected using a pass energy of 187.85 eV and a step size of 0.2 eV. All spectra were referenced to the binding energy of SnO_2 (486.6 eV), as Sn was expected to occur dominantly as SnO_2.

As Pd has been reported to reduce with X-ray exposure, the Pd 3d region was collected first for all scans. In addition, longer scans were performed after the initial scans were collected to exclude possible reduction. No shifts in peak position and shape in relation to the first scans were observed after these scans (Fig. S11).

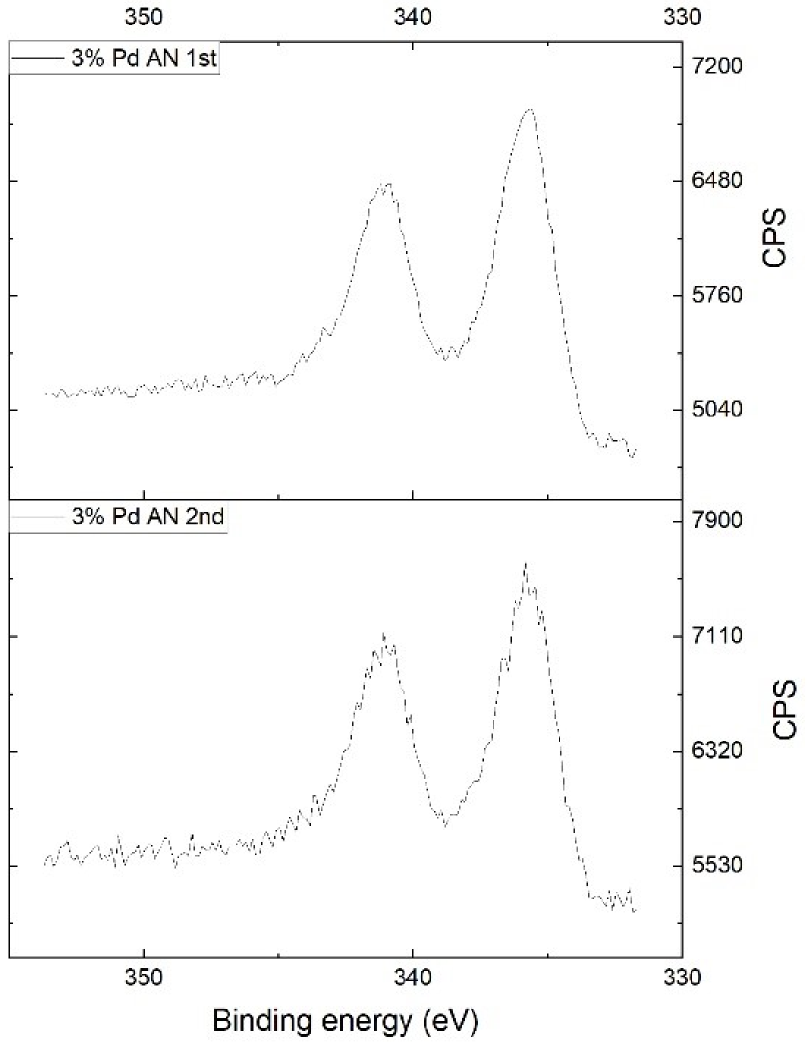

Fig. S11 Comparison of two XPS Pd 3d spectra taken after each other (upper graph = first scan, lower graph = second and longer scan).

The calculation of atomic concentrations of individual Pd species (Pd^0, PdO and PdO_2) was performed via curve fitting with CasaXPS software (Version 2.3.25PR1.0). A Shirley type background subtraction was performed. In addition, peak positions were set to the average of literature values (Table S3) and constrained to be within their individual spread (Table S3, min/max). Furthermore, the area ratio between Pd 3d 5/2 and 3/2 due to spin-orbit splitting was fixed at the literature ratio of 3:2[S1] and all peak position of the 3/2-component fixed at a ΔE of 5.26 eV relative to the 5/2 component.[S1] Peak profiles were considered to be a convolution of Gaussian and Lorentzian functions[21].

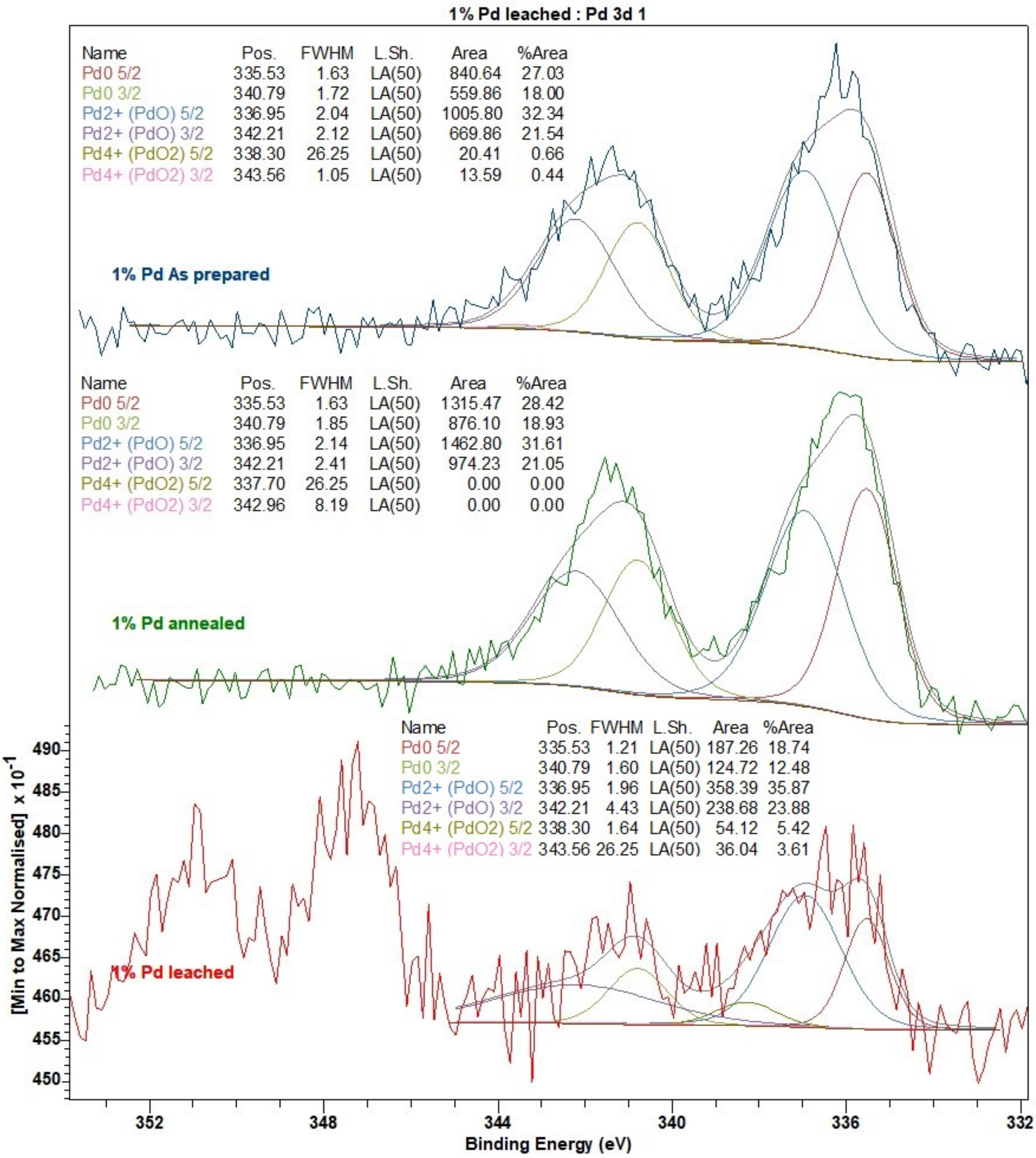

Fig. S12 XPS spectra and curve fitting from samples containing 1% Pd.

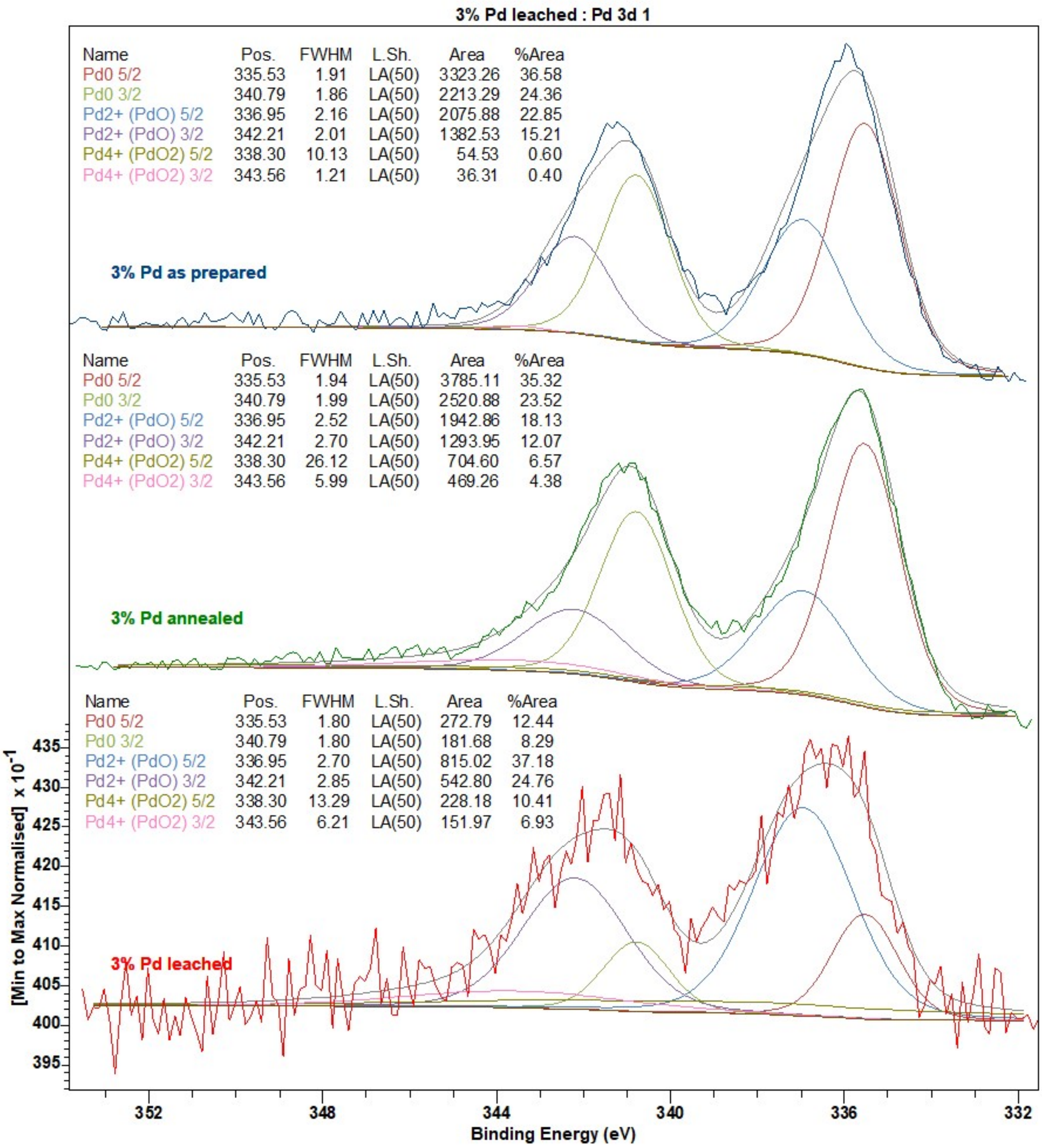

Fig. S13 XPS spectra and curve fitting from samples containing 3% Pd.

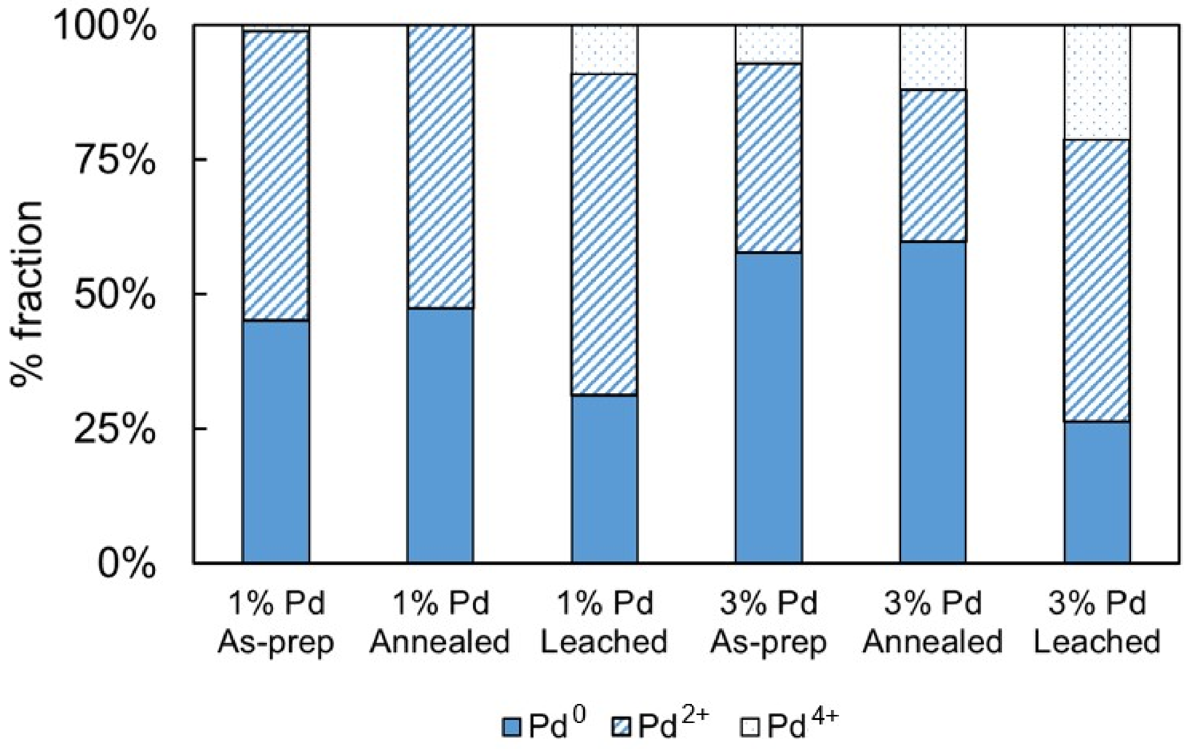

Fig. S14 Fraction of individual Pd species in the different treatments determined from curve fitting of the spectra in Fig. S12 and S13.

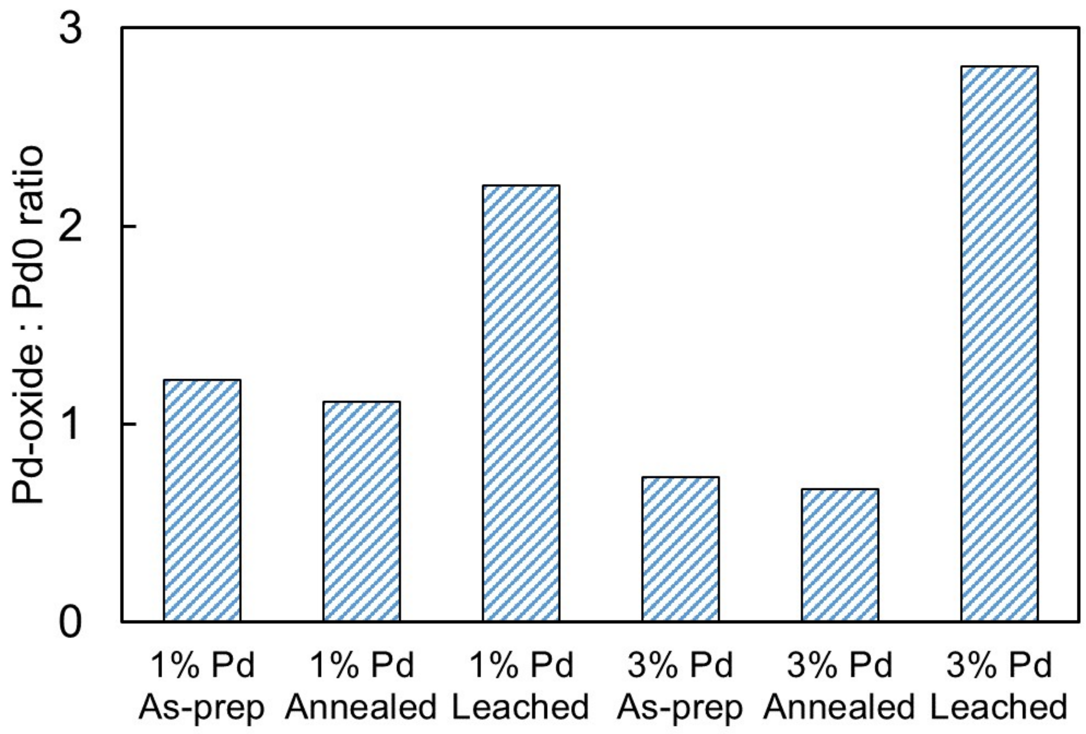

Fig. S15 Pd-oxide (sum of PdO and PdO_2) to Pd^0 ratio of the different treatments, determined from curve fitting of the spectra in Fig. S12 and S13.

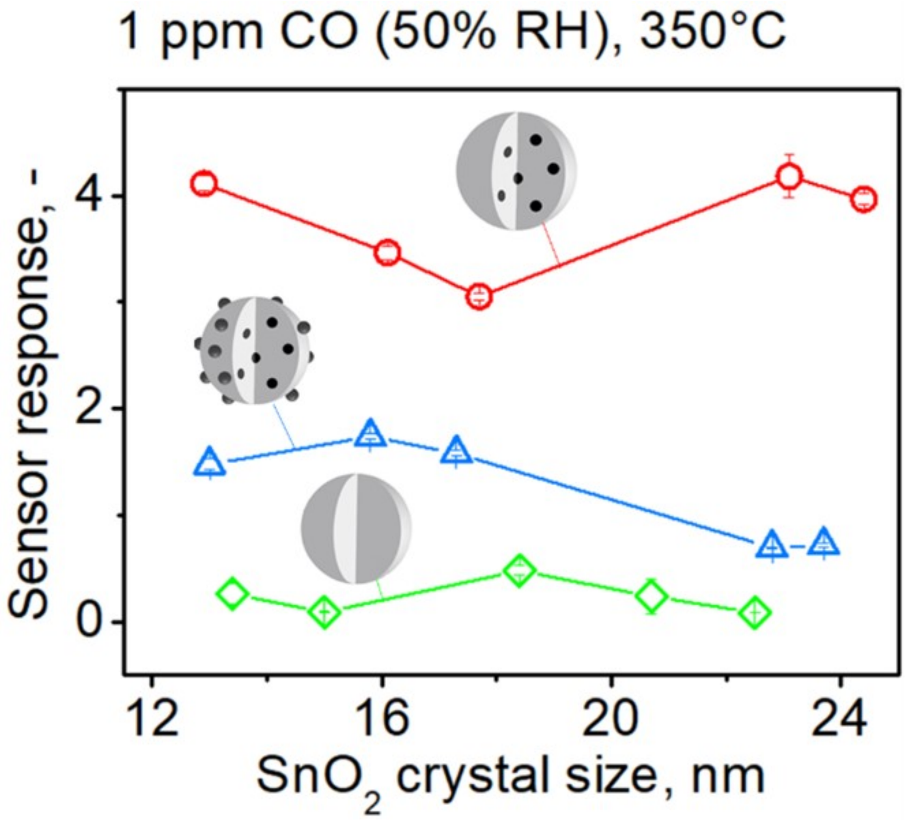

Fig. S16 The response to 1 ppm of CO at 350°C & 50% RH by sensors made with pure (green diamonds) and 0.2 mol% Pd-containing SnO_2 particles before (blue triangles) and after HNO_3 leaching and removal of Pd (red circles) made at various FSP precursor solution to dispersion O_2 flowrate ratio (starting from the left P/D = 1/5, 5/8, 5/5, 9/5, 5/2) and C = 1 M as a function of SnO_2 crystal size.

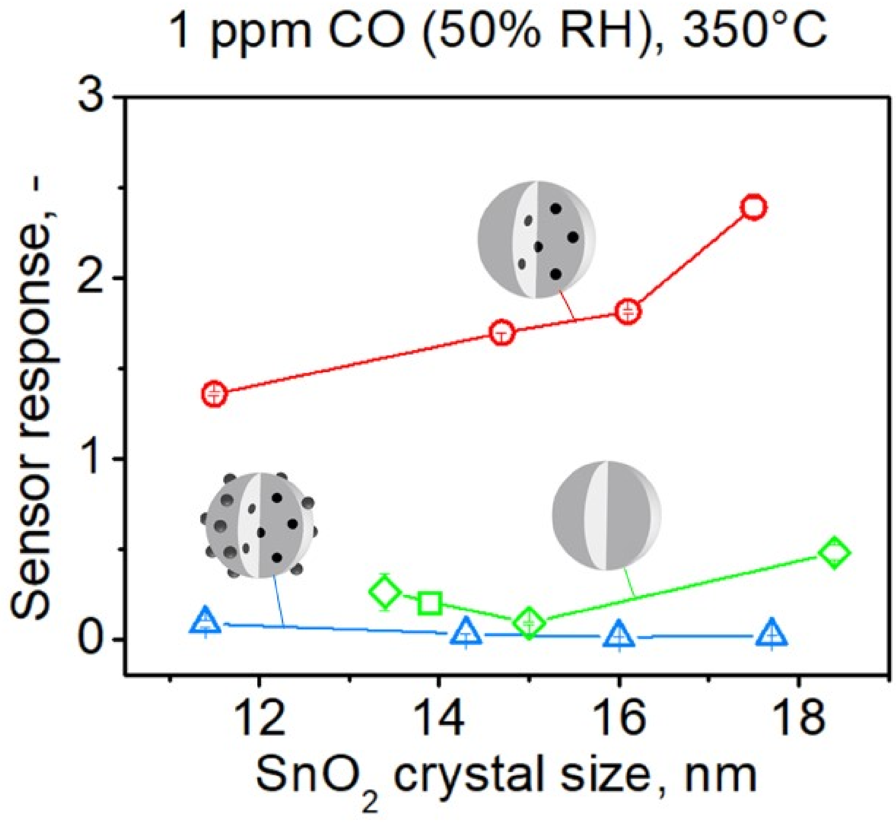

Fig. S17 The response to 1 ppm of CO at 350°C in 50% RH by pure (green square[13], and the diamonds – responses from Figure S14) and 1 mol% Pd-containing SnO_2 sensors before (blue triangles) and after HNO_3 solution leaching and removal of surface Pd (red circles) made at various FSP precursor solution concentration (starting from the left C = 0.1, 0.5, 1, 1.5 M) at P/D = 5/5. Please note that all Pd-containing sensor responses (with and without surface Pd) are below the corresponding ones made with 0.2 mol % Pd (Fig. S16). The sensors' response increases with increasing FSP precursor solution concentration C and SnO_2 crystal size (Fig. 1) and, most importantly, decreasing Pd content. For example, the sensor response nearly doubles from C = 0.1 M to C = 1.5 M as shown above but at the same time the embedded Pd content decreases from about 0.6 mol% to 0.4 mol% (Fig. S7).

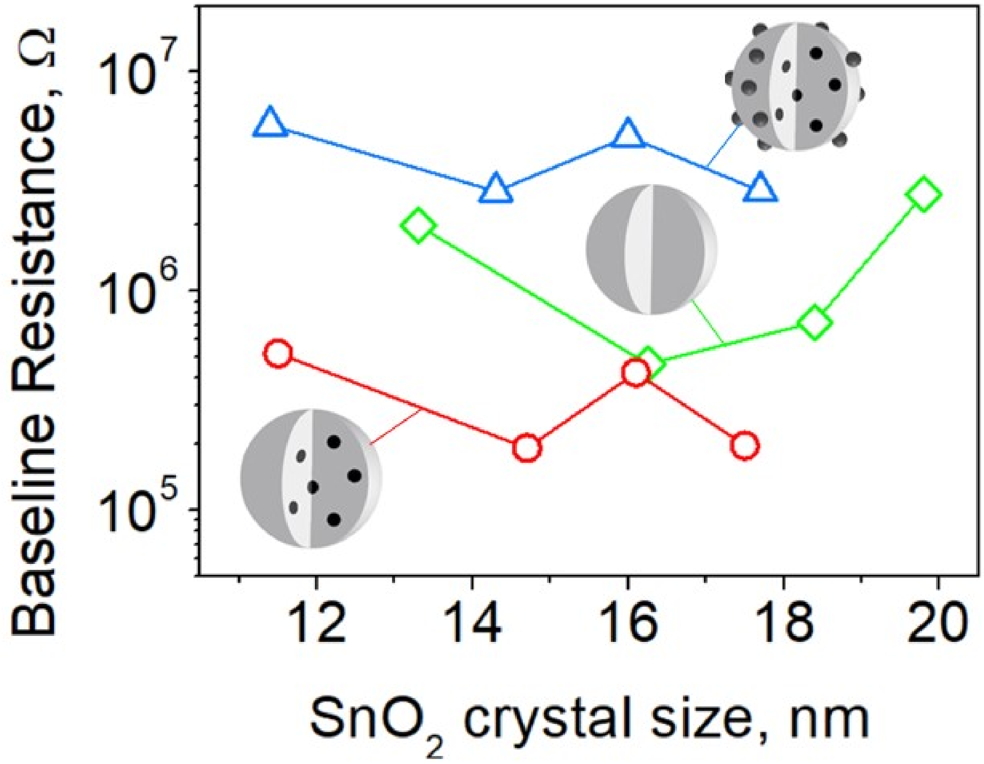

Fig. S18 Baseline resistance of pure and 0.2 mol% Pd-doped SnO_2 films as a function of SnO_2 crystal size for particles made at various precursor solution concentrations, C. As with Fig. 6, films of nanoparticles with only embedded Pd (red circles) have resistance significantly lower than the other two SnO_2 films.

Table S1 Table R1 Summary of selected Pd-containing SnO_2 sensors with the employed ranges of acetone and CO concentration ranges and operational variables (relative humidity, RH, and sensing temperature).

Material	Analyte	RH%	Operating Temp. °C	Concentration (ppm)	Ref.
Pd/SnO_2	Acetone	50	150-350	1	[11]
$Ce,Pd/SnO_2$	Acetone	30-60	150-325	10-1000	[15]
$Pd\text{-}SnO_2$	Acetone	n.A	RT	10-100	[17]
$Pd,Sm/SnO_2$	Acetone	n.A	100-450	5-500	[18]
Pd/SnO_2 leached	Acetone/CO	50	350	0 – 1	[13]
Pd/SnO_2	Acetone/CO	n.A	25-300	10-100	[16]
Pd/SnO_2	CO	n.A	20-400	100-1000	[19]
$Pd,Fe/SnO_2$	CO	n.A	150-400	200-3000	[20]
PdO_x SnO_2	CO	0-20	60-300	6.7	[21]
Pd/SnO_2	CO	n.A	200-400	50-240	[22]

Table S2 List of FSP synthesis process variables

Powder	Precursor solution conc.	Pd Loading	Nominal flows					Δp
			Precursor solution	Flamelet CH_4	Flamelet O_2	Disper. O_2	Sheath O_2	
	[M]	[mol%]	[mL/min]	[L/min]	[L/min]	[L/min]	[L/min]	[bar]
1% $PdSnO_2$ 0.1M 5/5	0.1	1	5	1.3	3.2	5	5	1.9
1% $PdSnO_2$ 0.5M 5/5	0.5	1	5	1.3	3.2	5	5	1.5
1% $PdSnO_2$ 1M 5/5	1	1	5	1.3	3.2	5	5	1.5
1% $PdSnO_2$ 1.5M 5/5	1.5	1	5	1.3	3.2	5	5	1.5
0.2% $PdSnO_2$ 0.1M	0.1	0.2	5	1.3	3.2	5	5	1.8
0.2% $PdSnO_2$ 0.5M	0.5	0.2	5	1.3	3.2	5	5	1.9
0.2% $PdSnO_2$ 1M 5/5	1	0.2	5	1.3	3.2	5	5	1.9
0.2% $PdSnO_2$ 1.5M	1.5	0.2	5	1.3	3.2	5	5	1.9
1% $PdSnO_2$ 0.5M 1/5	0.5	1	1	1.3	3.2	5	5	1.9
1% $PdSnO_2$ 0.5M 5/5	0.5	1	5	1.3	3.2	5	5	1.8
1% $PdSnO_2$ 0.5M 9/5	0.5	1	9	1.3	3.2	5	5	1.8
1% $PdSnO_2$ 0.5M 5/8	0.5	1	5	1.3	3.2	8	5	2.1
1% $PdSnO_2$ 0.5M 5/2	0.5	1	5	1.3	3.2	2	5	0.3
0.2% $PdSnO_2$ 1M 1/5	1	0.2	1	1.3	3.2	5	5	1.5
0.2% $PdSnO_2$ 1M 5/5	1	0.2	5	1.3	3.2	5	5	1.5
0.2% $PdSnO_2$ 1M 9/5	1	0.2	9	1.3	3.2	5	5	1.5
0.2% $PdSnO_2$ 1M 5/8	1	0.2	5	1.3	3.2	8	5	2.1
0.2% $PdSnO_2$ 1M 5/2	1	0.2	5	1.3	3.2	2	5	1.3
0.1% $PdSnO_2$ 0.5M	0.5	0.1	5	1.3	3.2	5	5	1.6
0.2% $PdSnO_2$ 0.5M	0.5	0.2	5	1.3	3.2	5	5	1.6
0.5% $PdSnO_2$ 0.5M	0.5	0.5	5	1.3	3.2	5	5	1.6
1% $PdSnO_2$ 0.5M 5/5	0.5	1	5	1.3	3.2	5	5	1.6
3% $PdSnO_2$ 0.5M 5/5	0.5	3	5	1.3	3.2	5	5	1.6
SnO_2 1M 1/5	1	0	1	1.3	3.2	5	5	1.5
SnO_2 1M 5/5	1	0	5	1.3	3.2	5	5	1.5
SnO_2 1M 9/5	1	0	9	1.3	3.2	5	5	1.5
SnO_2 1M 5/8	1	0	5	1.3	3.2	8	5	2.1
SnO_2 1M 5/2	1	0	5	1.3	3.2	2	5	1.3

Table S3 X-ray Photoelectron Spectroscopy (XPS): Literature peak positions for relevant Pd species in eV along with respective average, min/max and standard deviation values that were used to constrain the curve fitting model of XPS.

**Work cited**	**Pd^0**		**Pd^{2+} (PdO)**		**Pd^{4+} (PdO_2)**	
	3d 5/2	3d 3/2	3d 5/2	3d 3/2	3d 5/2	3d 3/2
Militello PdO[S2]			337.2	342.5		
Deligiannakis[21]	334.8	340.1				
Militello Pd^0 [S3]	335.2	340.5				
Lesiak[S4]	335.6	340	337	342	338	343.5
Gross[S5]	335.2		337			
XPS Handbook[S1]	335.2		336.3		337.9	
Average	335.2	340.2	336.875	342.25	337.95	343.5
Std. Dev.	0.28	0.26	0.4	0.35	0.07	
min	334.92	339.94	336.48	341.9	337.88	
max	335.48	340.46	337.27	342.6	338.02	

ESI exclusive references

S1 J.F. Moulder, W.F. Stickle, P.E. Sobol, K.D. Bomben, *Handbook of X-ray photoelectron spectroscopy,* 1992, Perkin Elmer Cooperation

S2 M. C. Militello, S. J. Simko, Palladium Oxide (PdO) by XPS. *Surf. Sci. Spectra,* 1994, **3**, 395–401, DOI: 10.1116/1.1247784

S3 M. C. Militello, S. J. Simko, Elemental Palladium by XPS. *Surf. Sci. Spectra,* 1994, **3**, 387–394, DOI: doi.org/10.1116/1.1247783

S4 B. Lesiak, L. Stobinski, L. Kövér, J. Tóth, K.J. Kurzydłowski, Ar ion bombardment modification of Pd–Au/MWCNTs catalyst surfaces studied by electron spectroscopy. *Phys. Status Solidi A,* 2011*,* **208**, 1791–1795, DOI: 10.1002/pssa.201001204

S5 S. Gross, L. Armelao, in: *X-ray Photoelectron Spectroscopy*, ed. J. M. Wagner, Nova Science Publishers, Inc., New York, 2011, Chapter 6: XPS as a powerful tool to investigate the surface properties of simple and mixed metal oxides, 125-186.
